# Iron, copper and disulfide dysregulation: molecular crossroads of metabolic cell death in melanoma progression

**DOI:** 10.3389/fphar.2025.1685331

**Published:** 2025-10-15

**Authors:** Xinge Li, Yan Gao, Zhiyao Xing, Zichuan Liu, Xiangyang Zhang

**Affiliations:** School of Pharmaceutical Science and Technology, Faculty of Medicine, Tianjin University, Tianjin, China

**Keywords:** melanoma, ferroptosis, cuproptosis, disulfidptosis, molecular interactions

## Abstract

Melanoma is a highly aggressive malignant tumor arising from melanocytes, with its incidence and mortality rates continuously rising in recent years, posing a major global public health challenge. Although traditional targeted therapies and immune checkpoint inhibitors have significantly improved survival in some patients, primary and acquired resistance remain common, creating an urgent need for new treatment strategies. In recent years, metabolic cell death, ferroptosis, cuproptosis, and disulfidptosis, has shown unique advantages in melanoma research. Ferroptosis directly kills tumor cells through iron-mediated lipid peroxidation; cuproptosis relies on copper-induced mitochondrial protein aggregation to inhibit tumor proliferation; disulfidptosis arises from disulfide stress caused by glucose deprivation. This review provides a detailed analysis on the mechanisms and metabolic competition paradoxes of these three types of metabolic cell death and integrates key metabolic nodes, such as related genes SLC7A11, GPX4, FDX1, LIPT1, and PPIC. Furthermore, we discuss innovative treatment strategies that significantly enhance therapeutic efficacy and overcome resistance, including the combination of metabolic cell death with immune cell regulation, nanoparticle delivery, and sonodynamic/photodynamic therapies. Ferroptosis, cuproptosis, and disulfidptosis each possess distinct advantages and characteristics in the context of melanoma development, metastasis, and drug resistance. Leveraging both their common and unique mechanisms offers new perspectives for improving treatment outcomes.

## 1 Introduction

Melanoma, a highly aggressive malignant tumor originating from melanocytes in the skin or other anatomical sites (e.g., ocular uvea, mucosa, meninges), has seen a persistent rise in global incidence and mortality, emerging as a major public health challenge. Epidemiological projections indicate 100,640 new cases and 8,290 deaths in the United States in 2024 (Long et al., 2023), with global incidence expected to 510,000 cases by 2040 (Ubellacker et al., 2020). Notably, the disease primarily affects Caucasian populations, with ultraviolet (UV) radiation exposure identified as the primary risk factor (Teixido et al., 2021). While early-stage localized melanoma is often curable through surgical resection (Tyrell et al., 2017), the prognosis for metastatic melanoma remains poor, with distant metastatic cases exhibiting a 5-year survival rate of less than one-third (Tao et al., 2025), underscoring the complexity of clinical management.

From a molecular pathological perspective, the aggressive nature of melanoma is closely linked to its mutation. Approximately 50%–60% of cases are linked to the BRAF V600E mutation (Anestopoulos et al., 2022; Marzagalli et al., 2019), together with other common mutations involving KRAS (Kirsten rat sarcoma viral oncogene homolog), NRAS (neuroblastoma RAS viral oncogene homolog), HRAS (Harvey rat sarcoma viral oncogene), CDKN2B (cyclin-dependent kinase inhibitor 2B), PTEN (phosphatase and tensin homolog), TERT (telomerase reverse transcriptase), and p53 (Ta et al., 2023). Especially, the metastatic potential of melanoma is associated with its unique metabolic adaptability: the lymphatic system promotes the distant dissemination of tumor cells by providing oxidative stress protection (Ubellacker et al., 2020). Additionally, the presence of immunosuppressive cells (e.g., regulatory T cells) in the tumor microenvironment (TME) and changes in the extracellular matrix are known to accelerate the progression of the disease (Xu et al., 2021).

Although targeted therapies and immunotherapies have significantly changed the treatment for advanced melanoma, their clinical applications still face multiple challenges. Compared with monotherapy, kinase inhibitors targeting BRAF mutations (e.g., vemurafenib, dabrafenib) combined with MEK inhibitors (e.g., trametinib) have improved median overall survival (OS) and progression-free survival (PFS) rates in patients with unresectable advanced metastatic BRAF-V600-mutant melanoma (Robert et al., 2019; Dhillon, 2016). Nevertheless, acquired resistance remains inevitable. 1mmune checkpoint inhibitors (ICIs), such as anti-PD-1 agents (pembrolizumab) and anti-CTLA-4 agents (ipilimumab), have achieved long-term survival in a subset of patients. However, a quite proportion of melanoma patients exhibit primary or acquired resistance (e.g., increased PD-L1 expression, impaired antigen presentation, or T-cell inactivation) resulting in 40%–65% failure in anti-PD-1 monotherapy and more than 70% failure in anti-CTLA-4 therapy (Gide et al., 2018). Additionally, chemotherapy and radiotherapy show limited effectiveness against metastatic melanoma and often lead to significant adverse effects.

Cell death is a fundamental biological process that maintains homeostasis in multicellular organisms. However, its dysregulation influences the pathogenesis and progression of diseases such as cancer (Hotchkiss et al., 2009). Traditionally, cell death is divided into two categories: non-regulated (accidental) cell death and regulated cell death. The former occurs passively due to external factors (e.g., physical injury or chemical toxicity) and lacks clear intracellular signaling pathways (Green and Victor, 2012). In contrast, regulated cell death is an orderly process governed by specific molecular programs. (Galluzzi et al., 2018; Tang et al., 2019).

Recent advances have revealed a distinct class of regulated cell death, which arises from metabolic imbalances caused by nutrient depletion or metal ion overload. This form of cell death–metabolic cell death–has often been referred to as “cell sabotage” (Green and Victor, 2012). It includes mechanisms like ferroptosis, cuproptosis, disulfidptosis, *etc.* (Mao et al., 2024). While the potential physiological roles of these “sabotage” mechanisms remain debated, a thorough analysis of their regulatory networks could lead to a better understanding of disease progression and the development of novel cancer therapies (Green and Victor, 2012). More and more emerging evidences have disclosed the pivotal role of copper, iron, and disulfide homeostasis in melanoma pathogenesis and therapeutic resistance. This review focuses on the molecular interplay in metabolic cell death including ferroptosis, cuproptosis and disulfidptosis. How dysregulation of these pathways leads to melanoma initiation, metastasis, and resistance to conventional therapies. We also evaluate their therapeutic potential, focusing on the interactions between metabolic stress and immune responses in melanoma, thereby providing critical insights for the development of novel therapeutic strategies.

## 2 Ferroptosis in melanoma: an iron-dependent cell death driven by lipid peroxidation

**FIGURE 1:**
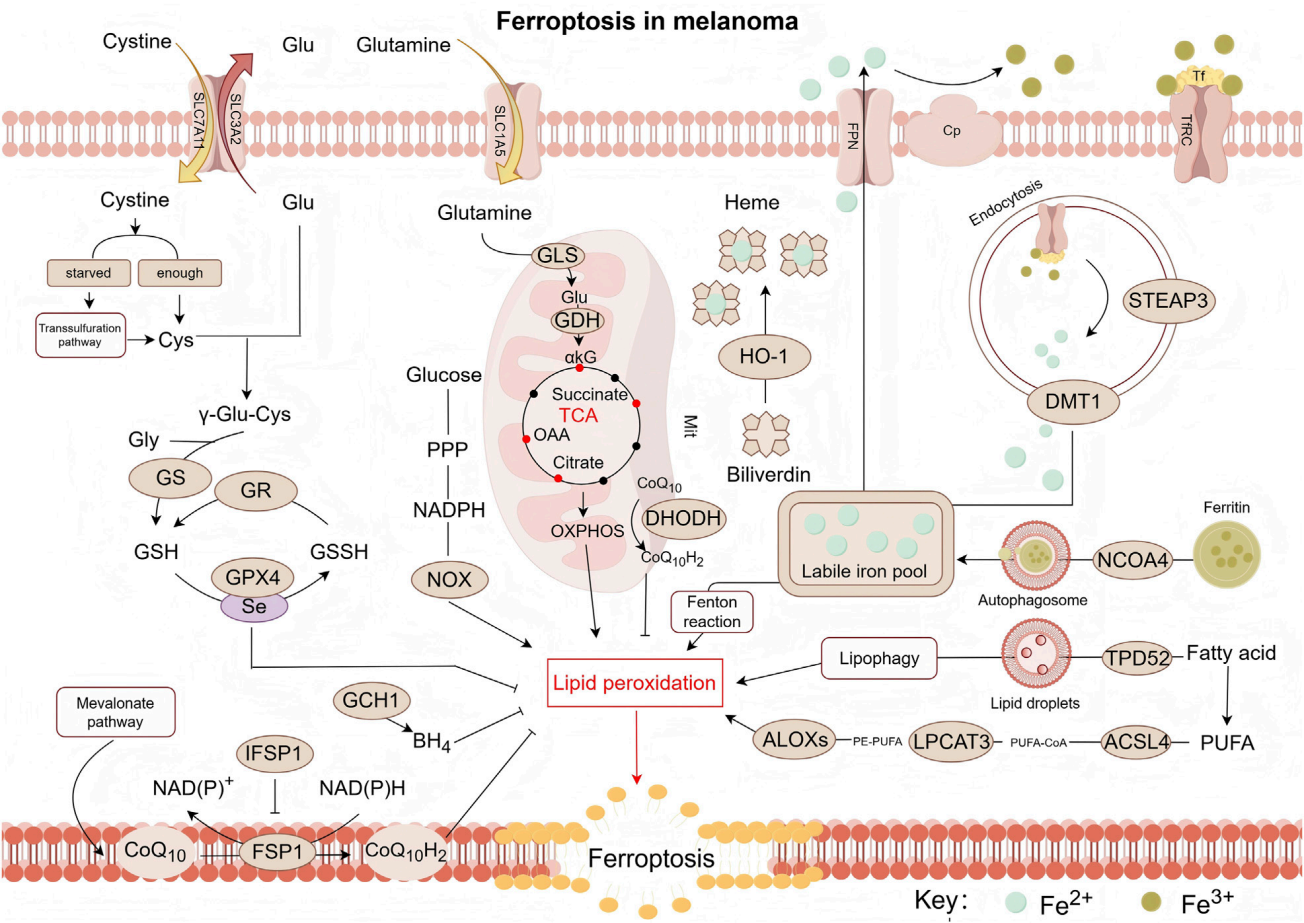
Pathway map of mechanisms associated with ferroptosis in melanoma. Ferroptosis is a form of cell death triggered by lipid peroxidation, which primarily occurs on polyunsaturated fatty acid-containing phospholipids (PUFA-PLs). Notably, PE-PUFA exhibit high susceptibility to ferroptosis. The synthesis of PE-PUFA is regulated by enzymes such as ACSL4, LPCAT3, and ALOXs. Ferroptosis relies on iron-mediated Fenton reactions, making the process highly sensitive to alterations in iron metabolism. This involves multiple steps including iron uptake (e.g., *via* transferrin), storage (e.g., *via* ferritin), release (e.g., *via* DMT1), and efflux (e.g., *via* ferroportin). To counteract lipid peroxidation and prevent ferroptosis, cells primarily depend on the GPX4 pathway as well as GPX4-independent pathways, such as those mediated by CoQ_10_H_2_, DHODH, and BH_4_—which constitute key antioxidant defense mechanisms. ROS, reactive oxygen species; Glu, glutamic acid; Cys, cysteine; GS, glutamine synthetase; GR, glutathione reductase; GSH, glutathione (Reduced); GSSH:glutathione (Oxidized); GPX4, glutathione peroxidase 4; GCH1, GTP cyclohydrolase 1; BH4:tetrahydrobiopterin; CoQ_10_, coenzyme Q10; CoQ_10_H_2_, reduced coenzyme Q10; FSP1, ferroptosis suppressor protein 1; IFSP1, ferroptosis suppressor protein 1; NOX, NADPH oxidase; PPP, pentose phosphate pathway; αkG, α-ketoglutaric acid; OAA, oxaloacetic acid; HO-1, Heme oxygenase 1; ALOXs, arachidonate lipoxygenases; LPCAT3, lysophosphatidylcholine Acyltransferase 3; DHODH, dihydroorotate dehydrogenase; ACSL4, acyl-CoA synthetase long-chain family member 4; TPD52, tumor protein D52; NCOA4, nuclear receptor coactivator 4; PUFA, polyunsaturated fatty acid; PE, phosphatidylethanolamine; CoA, coenzyme A; DMT1, divalent metal transporter 1; STEAP3, six-transmembrane epithelial antigen of prostate 3; Cp, ceruloplasmin; Tf, transferrin; TfRC, transferrin receptor; FPN/SLC40A1, ferroportin; SLC3A2, recombinant solute carrier family 3, member 2; SLC7A11, recombinant solute carrier family 7, member 11; ASCT2/SLC1A5, solute carrier family 1, member 5; GLS:glutaminase; GDH:glutamate dehydrogenase.

Thirteen years ago, Dixon’s lab proposed the concept of a unique form of regulated, iron-dependent cell death driven by lipid peroxidation, along with the term “Ferroptosis.” Three key research areas converged to establish the foundational understanding of the field of “ferroptosis”: (i) metabolic mechanisms, (ii) regulation of reactive oxygen species (ROS), and (iii) iron homeostasis (Stockwell, 2022), as illustrated in Figure 1.

### 2.1 Lipid metabolism and ferroptosis induction in melanoma

**FIGURE 2:**
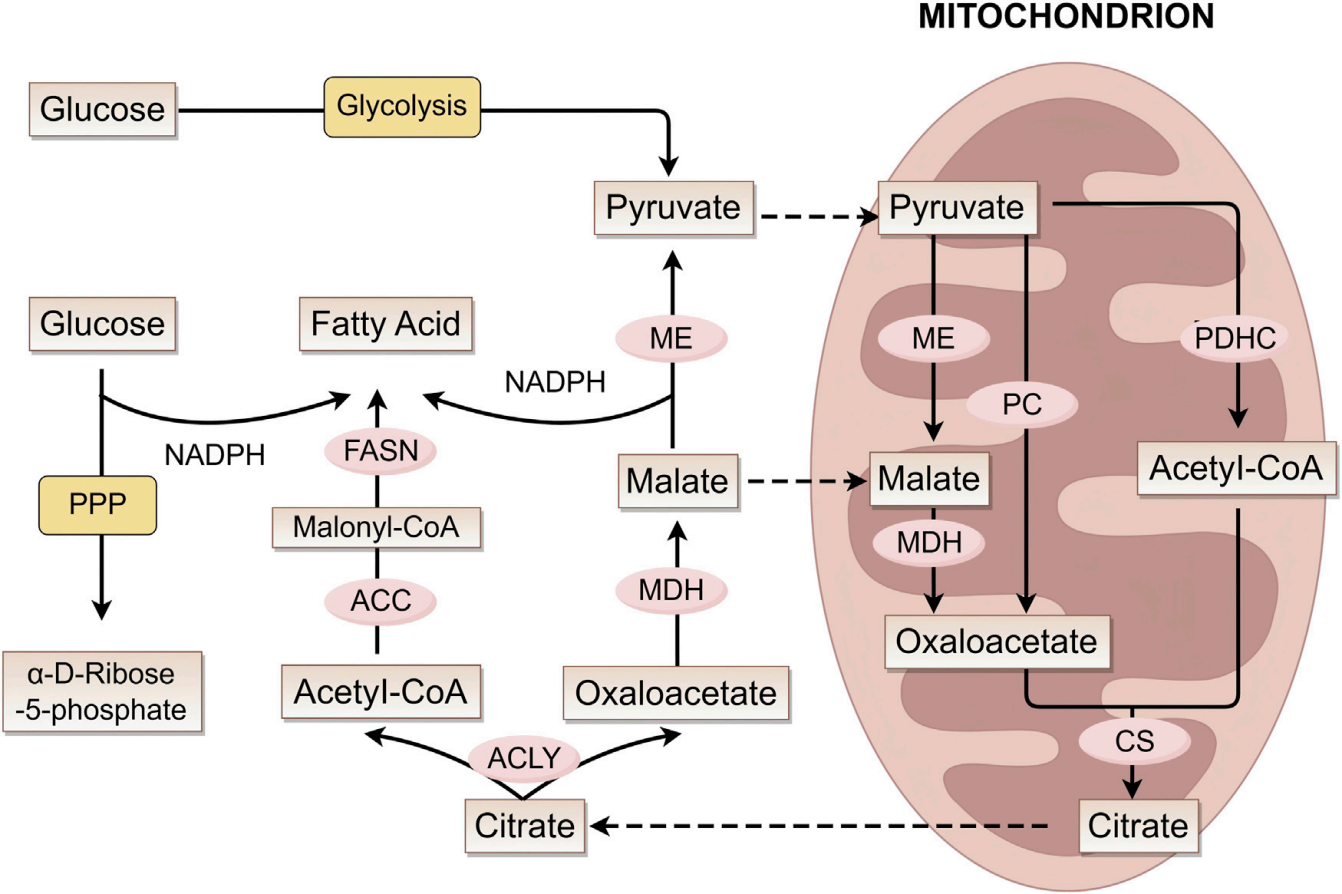
Fatty acid metabolic pathways and key enzymes. This figure illustrates the interconnected mechanisms of fatty acid synthesis, mitochondrial metabolism (involving the conversion of intermediate metabolites such as pyruvate, acetyl-CoA, and citrate), and glucose metabolic pathways. It demonstrates how these distinct metabolic processes are linked through key metabolites and enzymes (e.g., ME, FASN, ACLY), working in concert to coordinately regulate cellular material and energy metabolism. ME, malic enzyme; MDH, malate dehydrogenase; PC, pyruvate carboxylase; PDHC, pyruvate dehydrogenase complex; CS, citrate synthase; ACC, acetyl-CoA carboxylase; FASN, fatty acid synthase; ACLY, ATP citrate lyase.

Ferroptosis is closely linked to dysregulated lipid metabolism in melanoma, in which polyunsaturated fatty acid (PUFA) metabolism serves as the central molecular mechanism. Figure 2 shows the process of fatty acid formation in melanoma cells. Mitochondrial acetyl-CoA combines with oxaloacetate to form citrate *via* citrate synthase (CS) (Koundouros and Poulogiannis, 2020; Chhimpa et al., 2023), exported *via* citrate carrier (CIC). Notably, during metabolic stress such as hypoxia, the synthesis of acetyl-CoA preferentially originates from acetate. And melanoma, particularly brain metastases with poor prognosis, exhibits increased dependency on above-mentioned acetate, a weakness specific to BRAF-mutant tumors (Kamphorst et al., 2014; Mashimo et al., 2014; Lakhter et al., 2016). Cytosolic ATP-citrate lyase (ACLY) cleaves citrate into acetyl-CoA and oxaloacetate. Subsequent oxaloacetate conversion to malate (*via* MDH) and pyruvate (*via* ME) generates NADPH for biosynthesis (Simmen et al., 2020).

Fatty acid (FA) synthesis initiates with acetyl-CoA carboxylase (ACC) producing malonyl-CoA. Then, the fatty acid synthase (FASN) complex iteratively elongates the chain to produce palmitate (C16:0) (Wedan et al., 2024). Li and Kapur et al. discovered that ACC and FASN are significantly upregulated in human melanoma (Kapur et al., 2005; Li et al., 2016). Long-chain fatty acid (LCFA) elongation occurs mainly in the endoplasmic reticulum (ER), with desaturation being mediated by stearoyl-CoA desaturase (SCD) (Kubota and Espenshade, 2022). Research indicates that SCD activity may play a key role in melanoma phenotype switching: under low SCD conditions, ER stress is induced, suppresses microphthalmia-associated transcription factor (MITF), and promotes melanoma dedifferentiation (Vivas-García et al., 2020).

In melanoma ferroptosis, the oxidative modification of polyunsaturated PUFAs incorporated into membrane phospholipids plays a decisive role. Under oxidative stress, Reactive Oxygen Species (ROS) selectively attack hydrogen atoms from PUFA double bonds, triggering lipid peroxidation chain reactions (Yan et al., 2021). Studies reveal that phosphatidylethanolamine (PE)-bound PUFAs, particularly PE-AA and PE-AdA, exhibit marked sensitivity to ferroptosis (Fang et al., 2023), with their abundance positively correlating with acyl-CoA synthetase long-chain family member 4 (ACSL4) activity. Meanwhile, ACSL3, which belongs to the same family as ACSL4, is also associated with the poor prognosis of melanoma (Chen et al., 2016). One study indicates that the abundant oleic acid in lymphocytes protects melanoma cells from ferroptosis in an ACSL3-dependent manner. Notably, enzymatic oxidation of PUFAs is catalyzed by lipoxygenases (LOXs, particularly 15-LOX1/2) (Bouchaoui et al., 2023). This process is antagonized by ferroptosis inhibitors such as glutathione peroxidase 4 (GPX4), forming a key regulatory node that maintains redox balance in ferroptosis control.

### 2.2 GPX4 and its role in suppressing ferroptosis in melanoma

ROS, which include hydrogen peroxide (H_2_O_2_), hydroxyl radicals (·OH), singlet oxygen (^1^O_2_), and superoxide (·O_2_^−^), among others (Bedard and Krause, 2007), function as key signaling mediators in redox processes and are critically involved in both the initiation and progression of ferroptosis. The mitochondrial electron transport chain is considered major contributor to ROS generation. During coenzyme Q10 (CoQ_10_) mediated electron transfer, approximately 1%–2% of electrons leak during their transport from Complex I and II to Complex III (Gutierrez-Mariscal et al., 2021). Under specific conditions, these leaked electrons react with molecular oxygen to generate superoxide radicals (·O_2_^−^) (Parascandolo and Laukkanen, 2019), later converted to H_2_O_2_ by SOD. H_2_O_2_ can then react with free Fe^2+^ through the Fenton reaction, yielding highly reactive hydroxyl radicals (·OH). In BRAF-mutant melanoma, BRAF inhibitors upregulate oxidative phosphorylation (OXPHOS), enhancing mitochondrial ROS accumulation and cellular susceptibility to ferroptosis (Haq et al., 2013; Schöckel et al., 2015). Additionally, the NADPH oxidase (NOX) family specifically catalyzes the generation of O_2_^−^ *via* transmembrane electron transfer. NOX2 activation stimulates ROS production through a calcium signaling pathway dependent on the Ryanodine receptor (RyR) (Prosser et al., 2011).

The cystine/GSH/GPX4 axis represents a central defense mechanism against ferroptosis. System Xc^-^, which is a heterodimeric transmembrane transporter consisting of the light chain xCT (SLC7A11) and heavy chain 4F2hc (SLC3A2) (Koppula et al., 2018), facilitates the exchange of cystine and glutamate at a 1:1 ratio (Liu et al., 2021). It has been observed that BRAF inhibitor-resistant melanomas display increased reliance on glutamine and activate the NRF2 pathway, increasing xCT expression and GSH levels to evade ferroptosis (Khamari et al., 2018). Conversely, dedifferentiated melanomas are characterized by lower basal GSH levels and greater susceptibility to ferroptosis (Tsoi et al., 2018). Cystine within the cells is reduced to cysteine for glutathione (GSH) biosynthesis. GSH exists in reduced (GSH) and oxidized (GSSG) forms, dynamically balanced by glutathione peroxidase 4 (GPX4) and glutathione reductase (GR) (González-Domínguez et al., 2020). Among mammalian GPX family members (GPX1-GPX8), GPX4 is uniquely capable of clearing membrane lipid hydroperoxides. GSH biosynthesis is regulated in a stepwise manner: γ-glutamylcysteine ligase (GCL) catalyzes the conjugation of glutamate and cysteine to form γ-glutamylcysteine (γ-Glu-Cys) (Oppenheimer et al., 1979), followed by glycine addition *via* glutathione synthetase (GS) to produce GSH (Gasmi et al., 2024). In addition to System Xc^−^-mediated cystine uptake, mammalian cells utilize the transsulfuration pathway to derive cysteine from methionine. Studies indicate that elevated tumor microenvironmental methionine levels may disrupt GSH homeostasis through this pathway (Kamphorst et al., 2015).

### 2.3 Iron regulation and sensitivity to ferroptosis in melanoma

**FIGURE 3:**
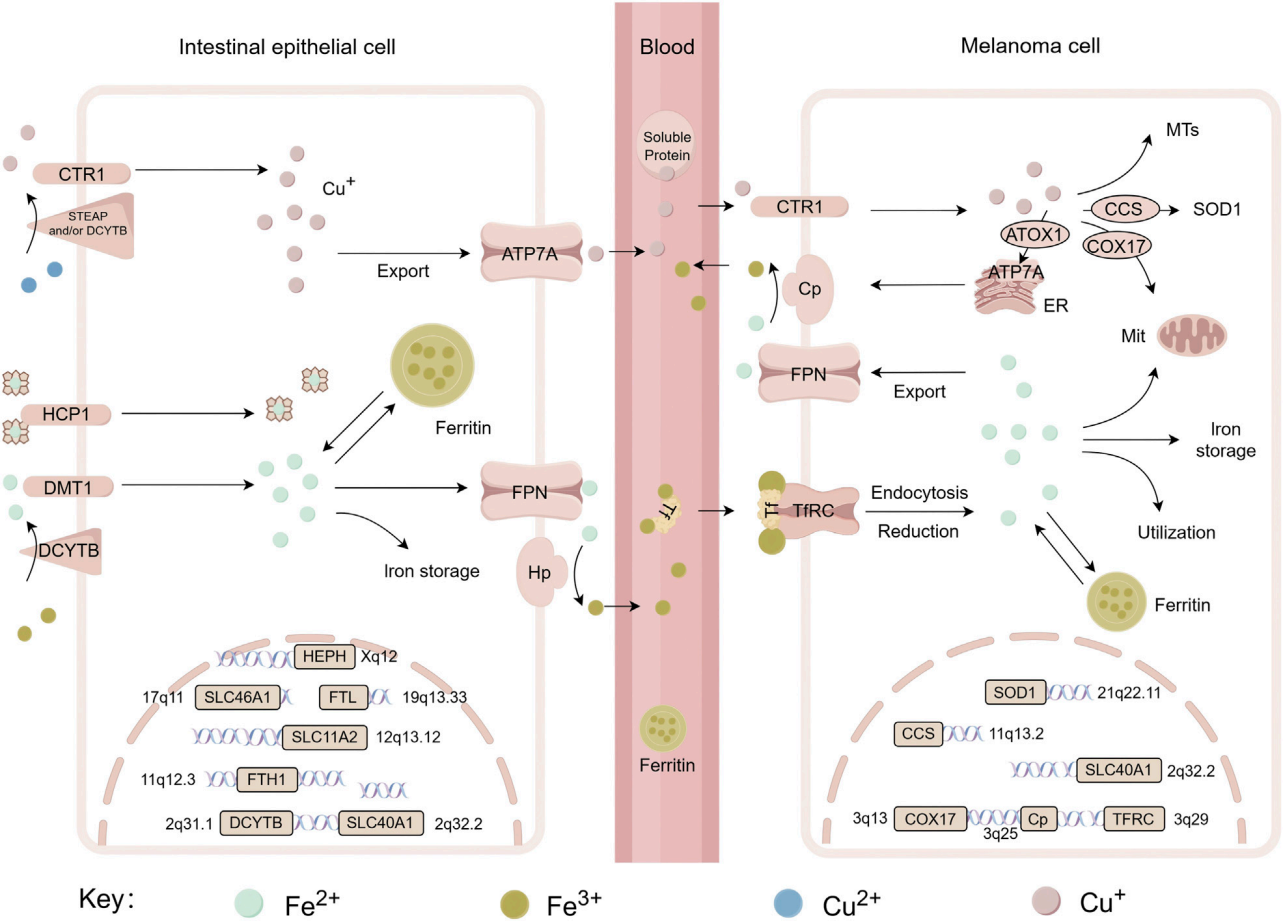
Systemic transport of iron and copper, related genes and their chromosomal localization. This figure shows the metabolic processes of iron (Fe) and copper (Cu) in intestinal epithelial cells and melanoma cells, including the uptake, transport, and storage of metal ions, as well as the roles of associated functional proteins such as CTR1, DMT1, Ferritin and so on. It also displays the chromosomal locations of key genes involved in these metabolic pathways—including SLC46A1, FTL, SOD1, *etc.*, thereby revealing the molecular mechanisms and regulatory gene networks underlying copper and iron metabolism across different cell types. DCYTB, duodenal cytochrome b; DMT1/SLC11A2, divalent metal transporter 1; HCP1/SLC46A1, heme carrier protein 1 (HCP1); STEAP, six-transmembrane epithelial antigen of prostate; CTR1/SLC31A1, copper transport protein 1; Hp/HEPH, hephaestin; FPN/SLC40A1, ferroportin; Cp, ceruloplasmin; FTL, ferritin light chain; FTH, ferritin heavy chain 1; TFRC, transferrin receptor; ATOX1, antioxidant 1; CCS, copper chaperone for superoxide dismutase; COX17, cytochrome c oxidase copper chaperone; SOD1, superoxide dismutase 1; ATP7A, copper-transporting ATPase 1; MTs, metallothioneins.

Iron, as an essential trace metal element in living organisms, participates in a wide range of critical biochemical processes, including oxygen transport, DNA synthesis and repair, and electron transfer in mitochondria (Galaris et al., 2019). From a chemical perspective, iron exhibits valence states ranging from −2 to +7. However, biological systems mainly utilize the +2 [ferrous, Fe (II)] and +3 [ferric, Fe (III)] redox states (Bayır et al., 2023). In melanoma, iron metabolism drives ferroptosis through multiple pathways. Heme iron is taken up by enterocytes *via* heme carrier protein 1 (HCP1) on their apical membrane as an intact porphyrin complex. Conversely, absorption of non-heme iron requires reduction of Fe^3+^ to Fe^2+^, which is catalyzed by duodenal cytochrome B (DCYTB), followed by transport across the membrane through divalent metal transporter 1 (DMT1/SLC11A2) (Dutt et al., 2022). Figure 3 illustrates the iron transport process, while the copper transport mechanism will be detailed in the next chapter.

Iron efflux from intestinal epithelial cells depends on the coordinated activity of ferroportin (FPN/SLC40A1) and hepcidin (Hp) at the basolateral membrane. Iron is extruded into the extracellular space by FPN, after which it is oxidized to Fe^3+^ by Hp, enabling its binding to transferrin (TF). The transferrin-iron complex in plasma is internalized by target cells *via* transferrin receptor (TfR)-mediated endocytosis (Tang et al., 2021). This process involves vesicular acidification, iron dissociation, reduction of Fe^3+^ catalyzed by STEAP3 (six-transmembrane epithelial antigen of the prostate 3), and final transport into the cytoplasm *via* DMT1.

In melanoma, the labile iron pool (LIP) expansion promotes Fenton reactions, which produce hydroxyl radicals that initiate lipid peroxidation, a key feature of ferroptosis. Excess iron is normally stored by ferritin, but ferritin degradation through ferritinophagy releases iron back into the pool, accelerating ferroptosis in melanoma (Gao et al., 2016). Crucially, iron regulatory protein 1 (IRP1) is upregulated by ferroptosis inducers (e.g., erastin, RSL3) in A375 and G361 melanoma cells, promoting ferroptosis by modulating TfR, FPN to increase intracellular iron (Yao et al., 2021).

Iron metabolism within the skin also plays a critical role in melanoma development. Disruption of iron balance induces oxidative stress in the skin microenvironment, accelerating inflammation and melanoma risk. Iron lack impairs skin metabolism, while excess iron promotes ferroptosis through ROS accumulation, representing a targetable weakness in melanoma treatment.

### 2.4 Alternative antioxidant pathways in ferroptosis resistance

Recent advancements in ferroptosis regulation research have revealed multiple novel antioxidant defense systems independent of the classic GSH-GPX4 pathway, offering new targeted strategies for cancer therapy. Non-GPX4 dependent pathways also play crucial roles in ferroptosis resistance, together constituting a defense system that operates across multiple cellular compartments.

Ferroptosis suppressor protein 1 (FSP1), primarily localized in lipid droplets and plasma membranes (Bersuker et al., 2018), acts as a key regulatory factor independent of the glutathione system. It uses NAD(P)H to reduce ubiquinone (CoQ_10_) to ubiquinol (CoQ_10_H_2_), which can directly neutralize lipid radicals and thus suppress lipid peroxidation (Bersuker et al., 2019; Doll et al., 2019). Emerging evidence indicates FSP1 can also exert ferroptosis resistance through mechanisms not involving CoQ10 by promoting membrane repair *via* the ESCRT-III complex (Dai et al., 2020). The STARD7 protein controls both the production and spatial distribution of CoQ10: its mitochondrial form synthesizes CoQ_10_, while the cytosolic form delivers it to various membranes, helping to form a trans-membrane antioxidant system (Deshwal et al., 2023).

Within mitochondria, DHODH and SQRDL constitute another protective layer. DHODH, a key enzyme in the pyrimidine synthesis pathway, reduces mitochondrial CoQ_10_ to CoQ_10_H_2_ and collaborating with mitochondrial GPX4 to combat mitochondrial specific lipid peroxidation. When GPX4 activity is compromised, DHODH maintains mitochondrial redox homeostasis through compensatory upregulation (Mao et al., 2021). SQRDL utilizes selenite as an electron donor to catalyze mitochondrial CoQ_10_ reduction. This pathway not only participates in antioxidant effects but also modulates ferroptosis susceptibility through mitochondrial electron transport chain regulation (Zarrinpar, 2022; Gui et al., 2021). It has been experimentally shown that combined inhibition of both DHODH and mitochondrial GPX4 induces extensive mitochondrial lipid peroxidation and irreversible ferroptosis, underscoring the functional synergy within this dual mitochondrial antioxidant system.

Furthermore, GCH1 (GTP cyclohydrolase 1), which is the rate-limiting enzyme for synthesizing tetrahydrobiopterin (BH4), inhibits ferroptosis through GPX4-independent mechanisms. As a potent free radical scavenger (Soula et al., 2020; Kraft et al., 2020), BH4 directly neutralizes lipid peroxidation radicals. In tumor cells lacking GPX4, the GCH1-BH4 pathway is often activated as a compensatory survival mechanism, becoming essential for maintaining cell viability.

## 3 Cuproptosis in melanoma: a mitochondrial damage activated by copper overload

In 2022, Tsvetkov et al. discovered a new type of regulated cell death that depends on copper and is induced by mitochondrial protein aggregation, which they named “cuproptosis”.

### 3.1 Dysregulated copper homeostasis promotes cuproptosis in melanoma

Both Systemic and cellular copper levels are tightly controlled to prevent toxicity. Dietary Cu^2+^ is reduced to Cu^+^ by STEAP or DCYTB and then imported through the high-affinity copper transporter CTR1 (SLC31A1) (Figure 3). CTR1 is overexpressed in melanoma biopsies compared to normal tissue (Mason, 1979; Lv et al., 2022; Georgatsou et al., 1997). DMT1 provides an alternative uptake when CTR1 is insufficient. Inside the cell, copper distribution is guided by specific chaperones: COX17 delivers copper to mitochondria (Horng et al., 2004; Cobine et al., 2006; Banci et al., 2008), CCS supplies copper to superoxide dismutase 1 (SOD1) for redox defense (Bertinato and L'Abbé, 2003; Prohaska et al., 2003), and ATOX1 is responsible for transferring copper to Cu-ATPases (ATP7A/B) in the trans-Golgi network (TGN) and supports the synthesis of cuproenzymes such as ceruloplasmin (Hamza et al., 2003). Meanwhile, the Cu-ATPases ATP7A (ubiquitous) and ATP7B (liver-specific) also act as the major transporters for exporting cellular copper (Lutsenko et al., 2007). Excess copper is stored by metallothioneins (MTs) or removed from the cell primarily through ATP7A and ATP7B (La Fontaine and Mercer, 2007; Palmgren and Nissen, 2011). Importantly, disruptions in copper export or storage trigger cytotoxic copper accumulation, directly linking cuproptosis in melanoma cells (Schmidt et al., 2018; Tsvetkov et al., 2022). Copper chelating active substance (e.g., D-penicillamine) induce phorbol-12-myristate-13-acetate-induced protein 1 (PMAIP1) expression, which upregulates NOXA protein, a necessary condition for melanoma cell death, highlighting the potential of targeting copper homeostasis as a therapeutic strategy (Qiao et al., 2012).

### 3.2 FDX1 and lipoylated proteins in cuproptosis activation

Distinct from ferroptosis, cuproptosis is directly triggered by copper overload and exhibits unique resistance to classical cell death inhibitors (Tsvetkov et al., 2022). Melanoma shows particular sensitivity to copper toxicity due to its metabolic characteristics: 35%–50% of wild-type, BRAF-mutant, and patient-derived melanoma cells rely heavily on oxidative phosphorylation (OXPHOS) for energy, making them sensitive to copper-induced mitochondrial damage (Fischer et al., 2018). The mechanism of cuproptosis is closely tied to mitochondrial function, as it can be suppressed *via* electron transport chain inhibitors (Tsvetkov et al., 2022). A key player is ferredoxin 1 (FDX1), which is highly expressed in melanoma and other cancers (Lu et al., 2023). FDX1 reduces Cu^2+^ to more toxic Cu^+^ and activates mitochondrial protein lipoylation by interacting with lipoic acid synthetase (LIAS) (Dreishpoon et al., 2023). Lipoylation, a post-translational modification, be disrupted during cuproptosis (Tsvetkov et al., 2022; Rowland et al., 2018). Notably, copper binds directly to lipoylated DLAT, triggering its oligomerization. This is thought to produce toxic protein aggregates that lead to cell death (Tsvetkov et al., 2022). Interestingly, high FDX1 expression in melanoma is linked to improved response to anti-PD-L1 immunotherapy (Lu et al., 2023), but FDX1 knockdown inhibits the *in vitro* proliferation of melanoma cells (Liu et al., 2022). Copper toxicity also damages iron-sulfur (Fe-S) cluster integrity. Recent studies have found that treating cells with a copper ionophore resulted in FDX1-dependent loss of Fe-S cluster proteins (Walshe, 2007), Figure 4 visually presents this process.

**FIGURE 4:**
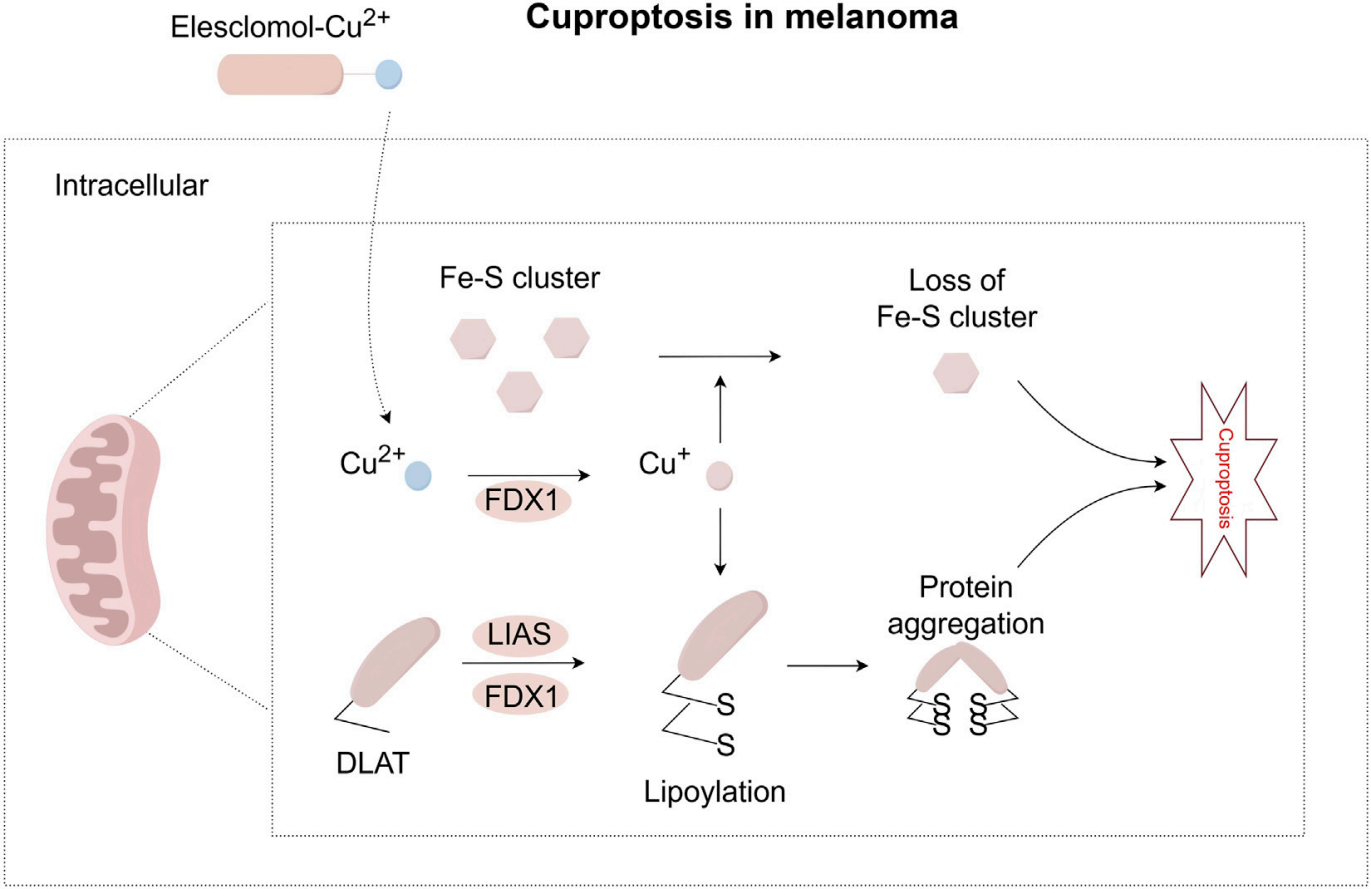
Pathway map of mechanisms associated with cuproptosis in melanoma. Extracellular copper enters cells through copper ionophores, such as elesclomol. Inside the cell, FDX1 reduces Cu^2+^ to Cu^+^. FDX1 also works with LIAS to support the lipoylation of certain metabolic enzymes, such as DLAT. When Cu^+^ binds directly to lipoylated proteins, it causes proteins to form oligomers. This abnormal process can lead to a toxic function that results in cuproptosis. At the same time, Cu^+^ can lead to the loss of iron-sulfur clusters in a process dependent on FDX1. These combined effects cause proteotoxic stress and eventually lead to cell death. FDX1, Ferredoxin 1; LIAS, Lipoic Acid Synthetase.

## 4 Disulfidptosis in melanoma: a novel cell death mechanism triggered by disulfide stress

**FIGURE 5:**
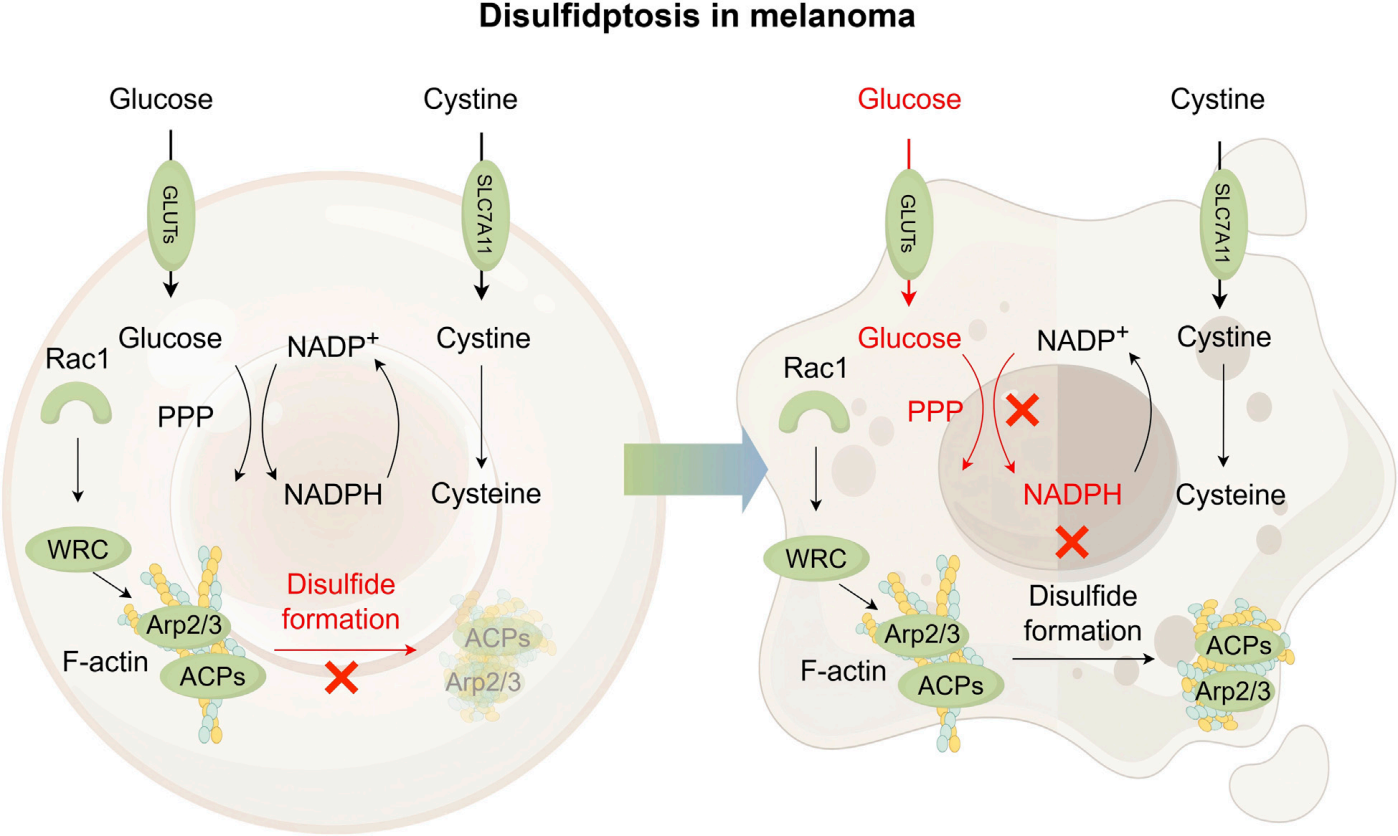
Pathway map of mechanisms associated with disulfidptosis in melanoma. In cells exhibiting high SLC7A11 expression, cystine uptake is significantly elevated, followed by its reduction to cysteine. This reduction process depends on NADPH as a key reducing agent, which is primarily supplied through the pentose phosphate pathway (PPP) using glucose. Abundant glucose is essential to prevent abnormal intracellular cystine accumulation and avoid disulfide bond formation within actin cytoskeleton proteins, thereby supporting cell survival in SLC7A11-high contexts. Under glucose-deficient conditions, limited NADPH availability leads to excessive buildup of cystine and other disulfide species. This promotes aberrant disulfide bonding in actin cytoskeletal proteins, resulting in the disruption of the actin network and ultimately inducing disulfidptosis. It is suggested that Rac1-WRC-Arp2/3-mediated branched actin polymerization may provide a structural environment conducive to disulfide bond formation among actin filaments, thereby promoting disulfidptosis. GLUT, glucose transporter; Rac1, Rac family small GTPase 1; ACPs, actin cytoskeleton proteins; WRC, Rac1-wave regulatory complex.

Disulfidptosis, identified in 2023, is a type of regulated cell death that occurs in cells with high SLC7A11 expression when glucose is scarce. Upon glucose starvation, cells with high expression of SLC7A11 experience rapid NADPH depletion and abnormal accumulation of insoluble disulfides, leading to disulfidptosis, as shown in Figure 5 (Liu et al., 2020; Goji et al., 2017; Liu X. et al., 2023; Joly et al., 2020). Importantly, this death mechanism differs from apoptosis, necroptosis and ferroptosis, as evidenced by insensitivity to their inhibitors and absence of classical markers like caspase-3 cleavage, cystine crystal formation or ATP depletion (Liu X. et al., 2023; Elmonem et al., 2016; Pereira et al., 2015). Cystine removal rescues these cells from glucose starvation-induced death (Liu et al., 2020; Goji et al., 2017), while thiol oxidizing agents worsen it (Liu X. et al., 2023). The glycolysis inhibitor 2-deoxyglucose (2DG) unexpectedly reduces cell death by shifting glucose analogs into the PPP to replenish NADPH (Liu et al., 2020; Zhang et al., 2014), confirming that NADPH supply not glycolytic is the key determinant. Disulfide-reducing agents (e.g., N-acetyl cysteine, tris (2-carboxyethyl) phosphine) restore NADPH levels and prevent cell death, further supporting disulfide overload as the main cause (Liu et al., 2020).

ROS scavengers show different effectiveness among various cell lines, indicating that ROS may influence but not initiate disulfidptosis (Liu et al., 2020; Joly et al., 2020). Further mechanistic insights reveal that glucose-starved cells with high expression of SLC7A11 leads to abnormal disulfide bonding within actin cytoskeleton proteins (ACPs), which results in the breakdown of the actin network, detachment of the plasma membrane, and ultimately disulfidptosis cell death (Liu D. et al., 2023). The Rac1-WAVE regulatory complex (WRC) pathway plays a critical role by activating Arp2/3-mediated branched actin polymerization and lamellipodia formation. This branched structure provides a site for disulfide bonding. Disulfidptosis is diminished upon WRC disruption and enhanced by Rac1 activation (Liu D. et al., 2023; Ibarra et al., 2005; Alekhina et al., 2017).

Correlatively, the movement and invasion of melanoma cells strongly rely on changes in the actin cytoskeleton. Although the WRC-Arp2/3 pathway is a main controller of this process, melanoma cells show a high level of flexibility. The co-activator YAP, which promotes melanoma growth and spread, controls the Arp2/3 subunit ARPC5. This subunit is essential for keeping focal adhesions stable and supporting the ability of melanoma cells to invade (Lui et al., 2022). This YAP-ARPC5 axis represents an alternative pathway influencing Arp2/3 activity and actin dynamics in melanoma. In studies using B16F1 melanoma cells, it was found that lamellipodia-like structures (LLS) driven by Arp2/3 and regulated by Rac/Cdc42 can form without the WRC. This suggests there is more complexity in how the actin network is controlled in melanoma cells (Kage et al., 2022). These results indicate that melanoma cells may have other or backup ways to reshape their actin structures, which might change how they respond to disulfidptosis caused by WRC activation.

## 5 Interactions between three metabolism-linked cell death pathways

Ferroptosis, cuproptosis, and disulfidptosis are programmed cell death pathways triggered by metabolic stress. They form a dynamic antagonistic-synergistic network through shared key molecular hubs. Table 1 provides a comparative analysis of their characteristics, while Table 2 lists common or potential inducers and inhibitors for these three forms of programmed cell death.

**TABLE 1:** Comparison of characteristics of ferroptosis, cuproptosis and disulfidptosis.

Feature	Ferroptosis	Cuproptosis	Disulfidptosis
Core Inducer	Iron ions (Fe^2+^)	Copper ions (Cu^+^)	Disulfide bonds
Morphological Features	Increased mitochondrial membrane density; Mitochondrial shrinkage; Reduction or disappearance of mitochondrial cristae; Outer membrane rupture (Li et al., 2023)	Mitochondrial shrinkage; Plasma membrane rupture; Endoplasmic reticulum damage (Tsvetkov et al., 2022)	Cell membrane contraction; loss of contents; collapse of the F-actin network; Cytoskeleton collapse (Liu et al., 2025)
Hallmark Metabolic Event	Lipid peroxidation of membrane phospholipid	Oligomerization of lipoylated TCA cycle proteins	Actin disulfide crosslinking
Key Defense Pathways	GPX4-GSH axis FSP1-CoQ10 axis DHODH	Copper chaperones (ATOX1, CCS)Copper efflux pumps (ATP7A/B)	NADPH regeneration
Death Executor	Accumulation of lipid ROS → Loss of membrane integrity	Mitochondrial protein aggregation → Proteotoxic stress	F-actin contraction → Plasma membrane detachment

**TABLE 2:** The effects of common or potential inhibitors and inducers of ferroptosis, cuproptosis and disulfidptosis in melanoma.

Category		Compound	Research Stage	Mechanism related to ferroptosis	Current application	Existing research on melanoma	Research material
Ferroptosis Inhibitors	ACSL4 Indirect Regulation	Rosiglitazone	Clinical use	PPARγ agonist; indirectly inhibits ACSL4 expression (Kung et al., 2022)	Type 2 diabetes	Promote the development of human melanoma in xenografts	A375 (Pich et al., 2018)
		Pioglitazone	Clinical use	PPARγ agonist; similar to Rosiglitazone (Kung et al., 2022)	Type 2 diabetes	Interfer TLR4-dependent signaling pathways to exert protective effects against melanoma	B16F10 (Dana et al., 2019)
	LOX Inhibition	Zileuton	Clinical use	Inhibits LOX activity; reducing lipid peroxidation (Costa et al., 2023)	Asthma treatment	—	—
	Antioxidant	Ferrostatin-1	Preclinical	Radicals scavenging; iron chelation (Guerrero-Hue et al., 2019)	Tool compound for mechanism studies	Inhibit melanoma ferroptosis	A375 (Tyurina et al., 2023)
		Liproxstatin-1	Preclinical	Clearing ROS; activates the Nrf2 pathway and restores GPX4 levels (Cao et al., 2021)	Tool compound for mechanism studies	Inhibit melanoma ferroptosis	M21 (Kim et al., 2016)
	ROS Scavenging	PHOXNO	Preclinical	Nitrogen oxide; blocks Fenton reaction and ROS generation (Xu et al., 2025)	Tool compound for mechanism studies	—	—
Ferroptosis Inducers	Targeting System Xc^-^	Erastin	Preclinical	Inhibits System Xc^−^; activates Nrf2/HO-1 pathway in cervical cancer cells (Dixon et al., 2012; Wei et al., 2023; Fishman et al., 2015)	Tool compound for mechanism studies	Induce melanoma ferroptosis	A375 G361 (Yao et al., 2021)
		Sorafenib	Clinical use	Inhibits System Xc^−^; suppresses BXIP/SCD axis in HCC (Dixon et al., 2012; Wei et al., 2023; Zhang et al., 2023a)	Advanced HCC, RCC	Induce melanoma ferroptosis	B16F10 (Yu et al., 2022)
		Sulfasalazine (SAS)	Clinical use	Inhibits System Xc^−^; increases Fe^2+^ and ROS levels in Neuroblastoma with MYCN amplification (Floros et al., 2021)	Inflammation	Decrease glutathione content, enhance susceptibility to radiation therapy	B16F10 (Nagane et al., 2018)
	Targeting GPX4	RSL3	Preclinical	Reduces GPX4 expression; increases ROS (Sui et al., 2018)	Tool compound for mechanism studies	Induce melanoma ferroptosis	A375 G361 (Yao et al., 2021)
		ML162	Preclinical	Inhibits GPX4; induces ROS accumulation (Zou et al., 2019)	Tool compound for mechanism studies	—	—
	Targeting CoQ10 Biosynthesis	iFSP1	Preclinical	Inhibits FSP1; reduces CoQ10 synthesis (Cheu et al., 2023)	Tool compound for mechanism studies	—	—
		Simvastatin	Clinical use	Inhibit HMGR, block MVA pathway and CoQ10/GPX4 biosynthesis in Triple-negative breast cancer (Xu et al., 2025)	Anti -hyperlipidemic drug	Combine simvastatin with tanshinone Ⅰ to regulate the expression of PARP1 and inhibit melanoma	A375 (Zhang et al., 2021)
	Targeting GSH	Cisplatin	Clinical use	Depletes GSH *via* Pt-GS complex formation; induces ferroptosis in NSCLC (Min et al., 2012)	Solid tumors	Anti melanoma	Patients with melanoma
		Acetaminophen (APAP)	Clinical use	Metabolite NAPQI depletes GSH; induces ferroptosis in hepatocytes (Lőrincz et al., 2015)	Pain/fever	—	—
	Targeting Fe^2+^/PUFA	Ferumoxytol	Clinical use	Increases Fe^2+^ levels (Rosner and Bolton, 2009; Birben et al., 2012)	Anemia in chronic kidney disease	Enhance immune effect of macrophages on melanoma	B16F10 (Stater et al., 2023)
		FINO2	Preclinical	Oxidizes iron, induces lipid peroxidation (Gaschler et al., 2018)	Tool compound for mechanism studies	—	—
		Salinomycin	Preclinical	Induce iron loading in the lysosomes, induces ROS (Zhao et al., 2018)	Broad-spectrum anti-bacterial agent; selective agent for cancer stem cells (CSCs)	Obviate liver metastasis of uveal melanoma	92.1 Mel270 Omm1 Omm2.3 (Zhou et al., 2019)
Cuproptosis Inhibitors	Targeting Cu^2+^	Ammonium tetrathiomolybdate	Phase II clinical trials	Copper chelating agents are also a type of sulfide donor (Chan et al., 2017)	Breast cancer	—	—
		Penicillamine	Preclinical	Copper chelating agents (Wang et al., 2023a)	Tool compound for mechanism studies	Induce Noxa (PMAIP1)-dependent mitochondrial apoptosis	A375 G361 (Qiao et al., 2012)
Cuproptosis Inducers	Targeting Cu^2+^	Disulfiram	Preclinical	The active form of DSF transports copper to intracellular compartments (Nie et al., 2022)	Tool compound for mechanism studies	Induce copper-dependent reactive oxygen species stimulation	B16F10 (Mohapatra et al., 2024)
		Elesclomol	Preclinical	Highly lipophilic copper ion carrier (Zheng et al., 2022)	Tool compound for mechanism studies	Load copper and induce cuproptosis	B16 (Lu et al., 2024)
		Zinc Pyrithione	Market exit	Copper ion carrier (Huo et al., 2023)	Antibacterial action	Increase the zinc content within melanoma cells and induce autophagy	Primary culture (Rudolf and Rudolf, 2021)
Disulfidptosis Inhibitors	Targeting System Xc^-^	Erastin	Preclinical	Block the cystine uptake mediated by SLC7A11 (Wang et al., 2024a)	Tool compound for mechanism studies	Induce ferroptosis (the main function)	—
	Targeting disulfide bond	DL-dithiothreitol	Preclinical	Maintain molecular structure (Yao et al., 2024)	Tool compound for mechanism studies	—	—
Disulfidptosis Inducers	Targeting System Xc^-^	Diethyl maleate	Preclinical	Upregulate the activity of system Xc^−^ (Deneke et al., 1989)	Tool compound for mechanism studies	—	—
	Targeting glucose	BAY-876	Preclinical	Glucose transporter 1 (GLUT1) inhibitor (Zhang et al., 2023b)	Tool compound for mechanism studies	—	—

### 5.1 The metabolic resource competition paradox among ferroptosis, cuproptosis, and disulfidptosis

Ferroptosis and disulfidptosis exhibit competitive regulatory features in NADPH generation and metabolic control. Ferroptosis relies on the enrichment of GSH precursors by system Xc^−^ and the antioxidant capacity of the GPX4-GSH system. In contrast, disulfidptosis arises from an imbalance between cystine intake driven by SLC7A11 and the use of NADPH. When glucose is limited, the PPP is unable to provide enough NADPH, which affects the activity of GPX4. The competition for metabolic resources between these two forms of cell death is notable. Specifically, high levels of SLC7A11 increase resistance to ferroptosis but also increase the risk of disulfidptosis due to excessive NADPH depletion. In addition, GPX4 also functions as an intracellular copper chelator to control copper levels inside the cell. Meanwhile, research has been shown that glutathione depletion induced by buthionine sulfoximine sensitizes cancer cells to cuproptosis (Mao et al., 2024; Tsvetkov et al., 2022). This competition at the metabolic level may help cells maintain a balance for cell fate selection.

### 5.2 Mitochondrial damage and disrupted metal homeostasis in ferroptosis, cuproptosis, and disulfidptosis

The interaction between cuproptosis and disulfidptosis mainly occurs at key points in mitochondrial metabolism. Copper ions induce abnormal aggregation of lipoylated DLAT in mitochondria *via* the FDX1-LIAS pathway, while NADPH deficiency in disulfidptosis may worsen mitochondrial function, forming a harmful cycle. The copper release mechanism involving ATP7A/B in cuproptosis shares similarities with the iron export process controlled by FPN in ferroptosis. At the same time, the stability of iron-sulfur clusters inside the cell affects whether cuproptosis is triggered, while ferroptosis also relies on Fe-S cluster biogenesis (Lee and Roh, 2023). This suggests that interfering with metal transport systems could impact multiple types of cell death simultaneously.

From an evolutionary perspective, the redox reactions involving metal ions naturally conflict with the precise control of cell metabolism especially in the tumor environment. To support rapid growth, cancer cells often accumulate metal ions in excess, but this makes them more sensitive to the activation of multiple death signals.

### 5.3 Molecular crosstalk among tumor microenvironment, ferroptosis, cuproptosis, and disulfidptosis

Ferroptosis, cuproptosis and disulfidptosis share certain common features in immune regulation, yet each exhibits distinct biological characteristics and mechanisms of action. Within the tumor microenvironment (TME), they collectively influence anti-tumor immune responses through metabolic reprogramming, immune cell polarization, and regulation of immune checkpoint expression.

The interaction between ferroptosis and the TME is complex. The sensitivity of immune cells to ferroptosis varies greatly. CD8^+^ T cells play a crucial role in triggering ferroptosis in cancer cells: IFNγ produced by CD8^+^ T cells suppresses the Xc^−^ system and upregulates ACSL4 expression, promoting ferroptosis in tumor cells (Dixon et al., 2012; Wang et al., 2019; Liao et al., 2022). However, some studies indicate that T cells themselves are also vulnerable to ferroptosis. Tumor-associated macrophages (TAMs) exhibit high plasticity and can differentiate into either immunostimulatory M1 or immunosuppressive M2 phenotypes. M2 macrophages, due to their lower antioxidant capacity, are more possible to ferroptosis than M1 macrophages (Kapralov et al., 2020; Luo et al., 2021). Inducing ferroptosis in M2 macrophages alleviates the immunosuppressive microenvironment, and modulating the M1/M2 macrophage ratio enhances the responsiveness to PD-1 therapy (Jiang et al., 2021), thereby improving the efficacy of cancer immunotherapy. NK cells also play a central role in anti-tumor immunity. NK cells inactivation in the TME are associated with oxidative stress, and activation of the transcription factor NRF2 has been shown to restore NK cells function (Sun et al., 2016; Poznanski et al., 2021). As the most potent antigen-presenting cells, dendritic cells (DCs) are essential for activating naive T cells and initiating T cell (Marmonti et al., 2022). Both the GPX4 inhibitor RSL3 and the lipid peroxidation product 4-hydroxynonenal (4-HNE) can impair dendritic cells (DCs) function (Cubillos-Ruiz et al., 2015). The effect of ferroptotic cancer cells on DCs may depend on the stage of ferroptosis. In early stages, cancer cells promote DCs maturation; however, as ferroptosis advances, this ability declines. Nonetheless, these late-stage ferroptotic cells remain susceptible to efficient phagocytosis by DCs (Efimova et al., 2020).

Cuproptosis modulates immune responses through copper-dependent mechanisms. Elevated copper levels in tumor tissues not only promote tumor proliferation and angiogenesis (Garber, 2015; Ge et al., 2022) but also specifically upregulate PD-L1 expression (Zhang et al., 2024), promoting immune escape. The copper transporter CTR1 is associated with infiltration of multiple immune cells (Wang J. et al., 2023) and correlates with poor prognosis in cancers such as breast cancer and melanoma (Lv et al., 2022; Wu et al., 2023). Copper chelators can reverse copper-induced immunosuppression and enhance infiltration of CD8^+^ T and NK cells (Cheng et al., 2022).

As a newly identified form of cell death, the direct link between disulfidptosis and the immune microenvironment remains unclear. However, its metabolic basis overlaps significantly with immune regulation. Key metabolites such as glucose, lactate, and cystine not only influence the occurrence of disulfidptosis but also strongly modulate immune cell function (Mi et al., 2024). For instance, high glucose consumption by tumor cells leads to nutrient deprivation in the TME and accumulation of lactate, which acidifies the TME. This acidic environment impairs T cell cytolytic activity and cytokine production (Apostolova and Pearce, 2022), while also promoting macrophage polarization toward the M2 phenotype and altering regulatory T cell (Treg) metabolism to sustain their function under low glucose conditions (Watson et al., 2021; Han et al., 2023). Furthermore, tumor cell expression of GLUT1 negatively correlates with CD8^+^ T cell infiltration. Inhibiting GLUT1 may not only induce disulfidptosis but also increase CD8^+^ T cell infiltration and reduce PD-L1 levels (Singer et al., 2011). Cystine deficiency similarly destroys T cell function (Wang et al., 2019), suggesting that modulating metabolite levels may together induce disulfidptosis and remodel the immune microenvironment.

These three forms of metabolic cell death interact with the TME through the release of and response to immune signaling molecules, as well as *via* metabolic competition. They share common involvement in metabolic reprogramming and immune checkpoint regulation, and all hold potential for combination with immunotherapy. Their distinctions lie in the fact that ferroptosis is closely linked to immune cell regulation, cuproptosis depends more on metal ion homeostasis and specific protein expression, while disulfidptosis is more intimately associated with glycolytic pathways. Current understanding of their immune effects is largely inferred from metabolic substrate competition, and specific mechanisms need further verification.

## 6 Therapeutic prospects of using metabolic cell death in melanoma

A complex network of molecular interactions and conflicting resource among ferroptosis, cuproptosis, and disulfidptosis creates multiple weakness in melanoma cells. However, effectively using these death mechanisms requires understanding the metabolic strategies of melanoma cells, especially when facing treatment resistance.

### 6.1 Strategies to reverse ferroptosis resistance in melanoma

Ferroptosis regulation in melanoma is closely related to changes in metabolism that lead to treatment resistance. Studies reveal that the unique lipid microenvironment of the lymphatic system protects metastatic melanoma cells from ferroptosis. Compared to blood, lymphatic fluid contains more glutathione and oleic acid. Oleic acid increases resistance to ferroptosis and supports the spread of cancer cells by activating the ACSL3-related signal (Ubellacker et al., 2020). BRAF inhibitor-resistant melanoma cells show changes in lipid metabolism, with lower levels of saturated fatty acids and higher levels of mono/polyunsaturated fatty acids. Targeting cholesterol esterification enzymes ACAT2 or SOAT helps regain drug sensitivity (Vergani et al., 2022). Transcriptional networks further enforce ferroptosis resistance in melanoma: EZH2 copy number amplification silences KLF14, upregulating SLC7A11 to enhance glutathione synthesis (Du et al., 2024), while APOE reduces polyunsaturated fatty acids and upregulates GPX4 (More et al., 2024). Resistance is similarly mediated by SREBP2-induced transferrin transcription, which reduces intracellular iron pools, ROS, and lipid peroxidation (Hong et al., 2021). These metabolic changes create a safe environment, allowing melanoma cells to avoid ferroptosis. Propafenone promotes mitochondrial HMOX1 expression by activating JNK/JUN signal, induces iron accumulation and ROS eruption, works together with RSL3 to promote ferroptosis in melanoma, Similarly, the CDK4/6 inhibitor palbociclib enhances the efficacy of the ferroptosis inducer auranofin by inducing cell senescence and depleting glutathione (GSH) and NADPH, This suggests that metabolic interventions as a strategy to overcome melanoma heterogeneity (Fan et al., 2024).

### 6.2 Amplify the therapeutic effect of melanoma by combined targeting of ferroptosis and immune activation

Multiple nanoplatforms combine the two functions of metabolic control and immune modulation. TPL@TFBF releases Fe^3+^ for Fenton reactions while inhibiting Nrf2, triggering ferroptosis/pyroptosis and releasing DAMPs to enhance CD8^+^ T cell infiltration (Wang S. et al., 2024). Similarly, the TCFI nanoplatform combines photodynamic therapy with ferroptosis to induce immunogenic cell death. Concurrently, interferon-γ secretion suppresses system xc^−^ activity, forming a positive environment between ferroptosis and antitumor immunity (Hou et al., 2023).

Post-translational modifications also provide potential targets to overcome resistance. Studies show that the balance between SUMOylation and phosphorylation of STAT1 is crucial for ferroptosis. A 108-amino acid polypeptide from circular RNA circPIAS1 interacts with the SUMO E3 ligase Ranbp2 to increase STAT1 SUMOylation, which in turn reduces its phosphorylation. This modification blocks the SLC7A11/GPX4 pathway and weakens interferon-γ-induced ferroptosis, ultimately leading to resistance against immune checkpoint inhibitors (ICB). This mechanism supports the idea of combining anti-circPIAS1 and PD-1 inhibitors in melanoma treatment (Zang et al., 2024). Another study identifies that the ubiquitin ligase Nedd4 induces resistance to ferroptosis by promoting VDAC2/3 degradation, a process inversely regulated by the FOXM1-Nedd4-VDAC2/3 (Yang et al., 2020). These research above suggest that using ferroptosis inducers together with agents that disrupt redox balance or promote immunogenic cell death can create combination therapies.

### 6.3 Innovative strategies for cuproptosis and disulfidptosis in melanoma treatment and the prognostic value of related genes

Cuproptosis and disulfidptosis are recently identified forms of metabolic cell death, but they show strong potential for melanoma therapy. Nanotechnology platforms are being developed to trigger tumor cell cuproptosis or disulfidptosis, aiming to improve treatment outcomes and solve shortcomings of traditional methods. For example, the nanocarrier ACM@MCHS-CuMOF@Dox, combining Mesoporous Carbon Hollow Spheres (MCHS) loaded with Copper-based Metal-Organic Frameworks (CuMOFs) and Doxorubicin (Dox), can downregulate FDX1 to induce apoptosis and cuproptosis, significantly inhibiting proliferation and migration of melanoma A375 cell line *in vitro* (Zhang et al., 2025); the triboelectric-field cuproptosis induction patch (TIP) utilizes a portable electric field to induce cuproptosis, overcoming limitations of traditional electrostimulation and effectively inhibiting postoperative melanoma recurrence (Chen et al., 2025). Another study developed a copper oxide nanoplateform (ES@CuO), which is absorbed by tumor cells and degrades to release Cu^2+^ triggering cuproptosis, significantly inhibiting B16 melanoma growth in mice, while promoting CD8^+^T cell infiltration and inflammatory factor secretion. Combining it with PD-1 immunotherapy further enhances the antitumor immune response (Lu et al., 2024); The nanoimmunoagonist pLCGM-OVA links cuproptosis with the cGAS-STING pathway to stimulate dendritic cell maturation and strengthen cytotoxic T lymphocyte activity against tumors (Li et al., 2025). Additionally, the ternary heterojunction (HACT) generates reactive oxygen species (ROS) and releases copper ions through sonodynamic therapy, inducing oxidative stress and cuproptosis while exhibiting high tumor-targeting specificity (Huang et al., 2024); drug-loaded nucleic acid nanomedicine (SNAMA) effectively inhibits tumor growth in primary and metastatic uveal melanoma models *via* GSH release and disulfidptosis activation (Tian et al., 2025).

Key genes associated with cuproptosis and disulfidptosis also exhibit clinical significance. Among cuproptosis-related genes (CRGs), LIPT1 is identified as an independent prognostic factor, positively correlating with PD-L1 expression while negatively regulating Treg cell infiltration (Lv et al., 2022); PPIC is a promoter of melanoma progression, enhancing cell invasiveness while suppressing CD8^+^T cell activation (Zhou et al., 2024). YAP1, a core gene in the Hippo pathway, positively correlates with FDX1 expression in the A2058 cell line, and impacts prognosis through modulation of M2 macrophage and Treg infiltration (Lv et al., 2024). Prognostic models constructed based on cuproptosis-related genes (CRGs) and disulfidptosis-related genes (DRGs) (e.g., CRSS score, lncRNA signature, 2-DRL prognostic model, *etc.*) enhance prediction accuracy, immunotherapy benefit rate, and tumor microenvironment (TME) status assessment capability (Liu D. et al., 2023; Cheng et al., 2024; Li et al., 2024; Lei et al., 2024; Zhao et al., 2023; Zhou et al., 2022; Yang et al., 2022; Zhu et al., 2024). These findings confirm that CRGs and DRGs have the potential to alter the TME and enhance treatment responsiveness, offering critical strategies for personalized precision medicine (Hu et al., 2023; Chen et al., 2022; Sun et al., 2023; Huang et al., 2023; Yang et al., 2024).

## 7 Conclusion and perspectives

Ferroptosis, cuproptosis, and disulfidptosis represent three key forms of metabolic cell death that offer novel therapeutic strategies for melanoma. This review highlights their dynamic metabolic competition network in melanoma cells, interconnected through mitochondrial metabolism and metal ion homeostasis regulation. For example, GPX4 depletion not only induces ferroptosis but also functions as an intracellular copper chelator, influencing cuproptosis initiation. Overexpression of SLC7A11 creates a paradoxical balance between ferroptosis resistance and disulfidptosis risk. Also, all three pathways profoundly affect the tumor microenvironment (TME). On the other hand, melanoma resistance mechanisms are closely linked to metabolic reprogramming, including the protection of the lipid microenvironment and the regulation of ACAT2/SOAT, SLC7A11, APOE, and other related genes. To address treatment resistance, the review integrates a variety of innovative anti-melanoma strategies that rely on ferroptosis, cuproptosis, and disulfidptosis, including nanoplatforms such as ES@CuO, TPL@TFBF, and ACM@MCHS-CuMOF@Dox. This review also identifies prognostic models with strong clinical predictive value, such as the CRSS score, lncRNA signature, and 2-DRL prognostic model. These findings suggest that precise regulation of metabolic cell death, redox balance, and interactions with immune checkpoints may overcome melanoma heterogeneity and drug-resistance bottlenecks.

Although these cell death mechanisms share common features, being induced by imbalances in intracellular metal ions or metabolic intermediates, and highlighting the complex interplay between cellular metabolism and death regulation, their research for melanoma therapy still faces significant limitations. Ferroptosis, being the earliest discovered among them, has relatively well-established detection methods, including lipid peroxidation probes, iron level assays, and glutathione metabolism markers. In contrast, detection methods for the more recently identified cuproptosis and disulfidptosis remain preliminary. Current approaches rely on copper ion measurement, FDX1/LIAS protein detection, F-actin morphology observation, SLC7A11 expression, and NADP+/NADPH ratio analysis. The lack of highly reliable and specific biomarkers severely limits accurate assessment and clinical application under pathological conditions, posing a major bottleneck for translational research.

Most existing studies are based on retrospective clinical data, and there is a lack of prospective experiments about melanoma cell sensitivity to different death, particularly under clinically relevant conditions such as untreated and treated (including resistant) conditions. Although metabolic cell death inducers such as copper ionophores (e.g., Elesclomol) and disulfiram have been studied in other cancers and non-cancer (e.g., NCT06635252, NCT05210374), confirming certain translational potential, no active clinical trials currently focus on melanoma patients.

Although targeting metabolic cell death has made some progress in melanoma, clinical translation still faces multiple challenges. Most inducers suffer from poor stability, low solubility, and limited bioavailability, which greatly restrict their clinical use. Developing novel inducers with better pharmacokinetic properties or repurposing FDA-approved anticancer drugs with cell death-inducing activity may partially solve these limitations. Nanomaterials, with their tunability, biocompatibility, and targeting capabilities, offer potential breakthroughs in melanoma treatment, though their actual efficacy and safety still require to be systematically verified. Furthermore, since different cell types within the tumor microenvironment exhibit different sensitivities to these death pathways, non-specific inducers or inhibitors may interfere with other cells, leading to experimental bias. Thus, the absence of cell-specific delivery strategies remains a major limitation.

Future research should focus on solving these critical gaps: first, deepening the mechanistic understanding of cuproptosis and disulfidptosis to clarify their molecular pathways and regulatory networks; second, developing highly specific and sensitive biomarker detection systems to support clinical diagnosis and treatment monitoring; and finally, innovating drug delivery strategies to improve targeting and safety. High-throughput functional screening and artificial intelligence approaches may accelerate the discovery of novel compounds targeting these three death modalities. Subsequent pharmacological studies should confirm their targeted delivery capabilities to enhance safety and efficacy. Targeting metabolic cell death holds promise as a new therapeutic approach in melanoma therapy, especially for patients who do not respond to conventional treatments. Ultimately, these efforts will improve our understanding of the mechanisms of cell death in melanoma and promote the clinical translation of related therapeutic strategies.

## References

[B1] Alekhina O. Burstein E. Billadeau D. D. (2017). Cellular functions of WASP family proteins at a glance. J. Cell Sci. 130 (14), 2235–2241. 10.1242/jcs.199570 28646090 PMC5536917

[B2] Anestopoulos I. Kyriakou S. Tragkola V. Paraskevaidis I. Tzika E. Mitsiogianni M. (2022). Targeting the epigenome in malignant melanoma: facts, challenges and therapeutic promises. Pharmacol. Ther. 240, 108301. 10.1016/j.pharmthera.2022.108301 36283453

[B3] Apostolova P. Pearce E. L. (2022). Lactic acid and lactate: revisiting the physiological roles in the tumor microenvironment. Trends Immunol. 43 (12), 969–977. 10.1016/j.it.2022.10.005 36319537 PMC10905416

[B4] Banci L. Bertini I. Ciofi-Baffoni S. Hadjiloi T. Martinelli M. Palumaa P. (2008). Mitochondrial copper(I) transfer from Cox17 to Sco1 is coupled to electron transfer. Proc. Natl. Acad. Sci. U. S. A. 105 (19), 6803–6808. 10.1073/pnas.0800019105 18458339 PMC2383975

[B5] Bayır H. Dixon S. J. Tyurina Y. Y. Kellum J. A. Kagan V. E. (2023). Ferroptotic mechanisms and therapeutic targeting of iron metabolism and lipid peroxidation in the kidney. Nat. Rev. Nephrol. 19 (5), 315–336. 10.1038/s41581-023-00689-x 36922653

[B6] Bedard K. Krause K. H. (2007). The NOX family of ROS-generating NADPH oxidases: physiology and pathophysiology. Physiol. Rev. 87 (1), 245–313. 10.1152/physrev.00044.2005 17237347

[B7] Bersuker K. Peterson C. W. H. To M. Sahl S. J. Savikhin V. Grossman E. A. (2018). A proximity labeling strategy provides insights into the composition and dynamics of lipid droplet proteomes. Dev. cell 44 (1), 97–112.e7. 10.1016/j.devcel.2017.11.020 29275994 PMC5764092

[B8] Bersuker K. Hendricks J. M. Li Z. Magtanong L. Ford B. Tang P. H. (2019). The CoQ oxidoreductase FSP1 acts parallel to GPX4 to inhibit ferroptosis. Nature 575 (7784), 688–692. 10.1038/s41586-019-1705-2 31634900 PMC6883167

[B9] Bertinato J. L'Abbé M. R. (2003). Copper modulates the degradation of copper chaperone for Cu,Zn superoxide dismutase by the 26 S proteosome. J. Biol. Chem. 278 (37), 35071–35078. 10.1074/jbc.M302242200 12832419

[B10] Birben E. Sahiner U. M. Sackesen C. Erzurum S. Kalayci O. (2012). Oxidative stress and antioxidant defense. World Allergy Organ. J. 5 (1), 9–19. 10.1097/WOX.0b013e3182439613 23268465 PMC3488923

[B11] Bouchaoui H. Mahoney-Sanchez L. Garçon G. Berdeaux O. Alleman L. Y. Devos D. (2023). ACSL4 and the lipoxygenases 15/15B are pivotal for ferroptosis induced by iron and PUFA dyshomeostasis in dopaminergic neurons. Free Radic. Biol. Med. 195, 145–157. 10.1016/j.freeradbiomed.2022.12.086 36581060

[B12] Cao Y. Li Y. He C. Yan F. Li J. R. Xu H. Z. (2021). Selective ferroptosis inhibitor liproxstatin-1 attenuates neurological deficits and neuroinflammation after subarachnoid hemorrhage. Neurosci. Bull. 37 (4), 535–549. 10.1007/s12264-020-00620-5 33421025 PMC8055759

[B13] Chan N. Willis A. Kornhauser N. Ward M. M. Lee S. B. Nackos E. (2017). Influencing the tumor microenvironment: a phase II study of copper depletion using tetrathiomolybdate in patients with breast cancer at high risk for recurrence and in preclinical models of lung metastases. Clin. Cancer Res. 23 (3), 666–676. 10.1158/1078-0432.Ccr-16-1326 27769988

[B14] Chen W. C. Wang C. Y. Hung Y. H. Weng T. Y. Yen M. C. Lai M. D. (2016). Systematic analysis of gene expression alterations and clinical outcomes for long-chain acyl-coenzyme A synthetase family in cancer. PloS One 11 (5), e0155660. 10.1371/journal.pone.0155660 27171439 PMC4865206

[B15] Chen Y. Chen X. Wang X. (2022). Identification of a prognostic model using cuproptosis-related genes in uveal melanoma. Front. Cell Dev. Biol. 10, 973073. 10.3389/fcell.2022.973073 36111345 PMC9468663

[B16] Chen W. Zhong S. Cai Q. Jiang Z. Hu Q. Tang C. (2025). A triboelectric-field-mediated cuproptosis induction patch for melanoma recurrence suppression. Matter 8 (5), 102088. 10.1016/j.matt.2025.102088

[B17] Cheng F. Peng G. Lu Y. Wang K. Ju Q. Ju Y. (2022). Relationship between copper and immunity: the potential role of copper in tumor immunity. Front. Oncol. 12, 1019153. 10.3389/fonc.2022.1019153 36419894 PMC9676660

[B18] Cheng S. Wang X. Yang S. Liang J. Song C. Zhu Q. (2024). Identification of novel disulfidptosis-related lncRNA signatures to predict the prognosis and immune microenvironment of skin cutaneous melanoma patients. Skin Res. Technol. 30 (7), e13814. 10.1111/srt.13814 38924611 PMC11197043

[B19] Cheu J. W. Lee D. Li Q. Goh C. C. Bao M. H. Yuen V. W. (2023). Ferroptosis suppressor protein 1 inhibition promotes tumor ferroptosis and anti-tumor immune responses in liver cancer. Cell. Mol. Gastroenterol. Hepatol. 16 (1), 133–159. 10.1016/j.jcmgh.2023.03.001 36893885 PMC10230009

[B20] Chhimpa N. Singh N. Puri N. Kayath H. P. (2023). The novel role of mitochondrial citrate synthase and citrate in the pathophysiology of Alzheimer’s disease. J. Alzheimer’s Dis. 94 (s1), S453–s472. 10.3233/jad-220514 37393492 PMC10473122

[B21] Cobine P. A. Pierrel F. Winge D. R. (2006). Copper trafficking to the mitochondrion and assembly of copper metalloenzymes. Biochimica Biophysica Acta 1763 (7), 759–772. 10.1016/j.bbamcr.2006.03.002 16631971

[B22] Costa I. Barbosa D. J. Benfeito S. Silva V. Chavarria D. Borges F. (2023). Molecular mechanisms of ferroptosis and their involvement in brain diseases. Pharmacol. Ther. 244, 108373. 10.1016/j.pharmthera.2023.108373 36894028

[B23] Cubillos-Ruiz J. R. Silberman P. C. Rutkowski M. R. Chopra S. Perales-Puchalt A. Song M. (2015). ER stress sensor XBP1 controls anti-tumor immunity by disrupting dendritic cell homeostasis. Cell 161 (7), 1527–1538. 10.1016/j.cell.2015.05.025 26073941 PMC4580135

[B24] Dai E. Zhang W. Cong D. Kang R. Wang J. Tang D. (2020). AIFM2 blocks ferroptosis independent of ubiquinol metabolism. Biochem. Biophys. Res. Commun. 523 (4), 966–971. 10.1016/j.bbrc.2020.01.066 31964528

[B25] Dana N. Vaseghi G. Haghjooy Javanmard S. (2019). PPAR γ agonist, pioglitazone, suppresses melanoma cancer in mice by inhibiting TLR4 signaling. J. Pharm. Pharm. Sci. 22 (1), 418–423. 10.18433/jpps30626 31509504

[B26] Deneke S. M. Baxter D. F. Phelps D. T. Fanburg B. L. (1989). Increase in endothelial cell glutathione and precursor amino acid uptake by diethyl maleate and hyperoxia. Am. J. Physiol. 257 (4 Pt 1), L265–L271. 10.1152/ajplung.1989.257.4.L265 2801955

[B27] Deshwal S. Onishi M. Tatsuta T. Bartsch T. Cors E. Ried K. (2023). Mitochondria regulate intracellular coenzyme Q transport and ferroptotic resistance *via* STARD7. Nat. Cell Biol. 25 (2), 246–257. 10.1038/s41556-022-01071-y 36658222 PMC9928583

[B28] Dhillon S. (2016). Dabrafenib plus trametinib: a review in advanced melanoma with a BRAF (V600) mutation. Target. Oncol. 11 (3), 417–428. 10.1007/s11523-016-0443-8 27246822

[B29] Dixon S. J. Lemberg K. M. Lamprecht M. R. Skouta R. Zaitsev E. M. Gleason C. E. (2012). Ferroptosis: an iron-dependent form of nonapoptotic cell death. Cell 149 (5), 1060–1072. 10.1016/j.cell.2012.03.042 22632970 PMC3367386

[B30] Doll S. Freitas F. P. Shah R. Aldrovandi M. da Silva M. C. Ingold I. (2019). FSP1 is a glutathione-independent ferroptosis suppressor. Nature 575 (7784), 693–698. 10.1038/s41586-019-1707-0 31634899

[B31] Dreishpoon M. B. Bick N. R. Petrova B. Warui D. M. Cameron A. Booker S. J. (2023). FDX1 regulates cellular protein lipoylation through direct binding to LIAS. J. Biol. Chem. 299 (9), 105046. 10.1016/j.jbc.2023.105046 37453661 PMC10462841

[B32] Du H. Hou L. Yu H. Zhang F. Tong K. Wu X. (2024). Enhancer of zeste homolog 2 protects mucosal melanoma from ferroptosis *via* the KLF14-SLC7A11 signaling pathway. Cancers 16 (21), 3660. 10.3390/cancers16213660 39518098 PMC11545276

[B33] Dutt S. Hamza I. Bartnikas T. B. (2022). Molecular mechanisms of iron and heme metabolism. Annu. Rev. Nutr. 42, 311–335. 10.1146/annurev-nutr-062320-112625 35508203 PMC9398995

[B34] Efimova I. Catanzaro E. Van der Meeren L. Turubanova V. D. Hammad H. Mishchenko T. A. (2020). Vaccination with early ferroptotic cancer cells induces efficient antitumor immunity. J. Immunother. Cancer 8 (2), e001369. 10.1136/jitc-2020-001369 33188036 PMC7668384

[B35] Elmonem M. A. Veys K. R. Soliman N. A. van Dyck M. van den Heuvel L. P. Levtchenko E. (2016). Cystinosis: a review. Orphanet J. Rare Dis. 11, 47. 10.1186/s13023-016-0426-y 27102039 PMC4841061

[B36] Fan L. Du P. Li Y. Chen X. Liu F. Liu Y. (2024). Targeted liposomes sensitize plastic melanoma to ferroptosis *via* senescence induction and coenzyme depletion. ACS Nano. 18 (9), 7011–7023. 10.1021/acsnano.3c10142 38390865

[B37] Fang X. Ardehali H. Min J. Wang F. (2023). The molecular and metabolic landscape of iron and ferroptosis in cardiovascular disease. Nat. Rev. Cardiol. 20 (1), 7–23. 10.1038/s41569-022-00735-4 35788564 PMC9252571

[B38] Fischer G. M. Vashisht Gopal Y. N. McQuade J. L. Peng W. DeBerardinis R. J. Davies M. A. (2018). Metabolic strategies of melanoma cells: mechanisms, interactions with the tumor microenvironment, and therapeutic implications. Pigment Cell Melanoma Res. 31 (1), 11–30. 10.1111/pcmr.12661 29049843 PMC5742019

[B39] Fishman M. N. Tomshine J. Fulp W. J. Foreman P. K. (2015). A systematic review of the efficacy and safety experience reported for sorafenib in advanced renal cell carcinoma (RCC) in the post-approval setting. PloS One 10 (4), e0120877. 10.1371/journal.pone.0120877 25830512 PMC4382117

[B40] Floros K. V. Cai J. Jacob S. Kurupi R. Fairchild C. K. Shende M. (2021). MYCN-amplified neuroblastoma is addicted to iron and vulnerable to inhibition of the system Xc-/glutathione axis. Cancer Res. 81 (7), 1896–1908. 10.1158/0008-5472.Can-20-1641 33483374 PMC9281612

[B41] Galaris D. Barbouti A. Pantopoulos K. (2019). Iron homeostasis and oxidative stress: an intimate relationship. Biochimica Biophys. Acta Mol. Cell Res. 1866 (12), 118535. 10.1016/j.bbamcr.2019.118535 31446062

[B42] Galluzzi L. Vitale I. Aaronson S. A. Abrams J. M. Adam D. Agostinis P. (2018). Molecular mechanisms of cell death: recommendations of the nomenclature committee on cell death 2018. Cell Death Differ. 25 (3), 486–541. 10.1038/s41418-017-0012-4 29362479 PMC5864239

[B43] Gao M. Monian P. Pan Q. Zhang W. Xiang J. Jiang X. (2016). Ferroptosis is an autophagic cell death process. Cell Res. 26 (9), 1021–1032. 10.1038/cr.2016.95 27514700 PMC5034113

[B44] Garber K. (2015). Cancer's copper connections. Sci. (New York, NY) 349 (6244), 129. 10.1126/science.349.6244.129 26160924

[B45] Gaschler M. M. Andia A. A. Liu H. Csuka J. M. Hurlocker B. Vaiana C. A. (2018). FINO(2) initiates ferroptosis through GPX4 inactivation and iron oxidation. Nat. Chem. Biol. 14 (5), 507–515. 10.1038/s41589-018-0031-6 29610484 PMC5899674

[B46] Gasmi A. Nasreen A. Lenchyk L. Lysiuk R. Peana M. Shapovalova N. (2024). An update on glutathione's biosynthesis, metabolism, functions, and medicinal purposes. Curr. Med. Chem. 31 (29), 4579–4601. 10.2174/0109298673251025230919105818 37921175

[B47] Ge E. J. Bush A. I. Casini A. Cobine P. A. Cross J. R. DeNicola G. M. (2022). Connecting copper and cancer: from transition metal signalling to metalloplasia. Nat. Rev. Cancer 22 (2), 102–113. 10.1038/s41568-021-00417-2 34764459 PMC8810673

[B48] Georgatsou E. Mavrogiannis L. A. Fragiadakis G. S. Alexandraki D. (1997). The yeast Fre1p/Fre2p cupric reductases facilitate copper uptake and are regulated by the copper-modulated Mac1p activator. J. Biol. Chem. 272 (21), 13786–13792. 10.1074/jbc.272.21.13786 9153234

[B49] Gide T. N. Wilmott J. S. Scolyer R. A. Long G. V. (2018). Primary and acquired resistance to immune checkpoint inhibitors in metastatic melanoma. Clin. Cancer Res. 24 (6), 1260–1270. 10.1158/1078-0432.Ccr-17-2267 29127120

[B50] Goji T. Takahara K. Negishi M. Katoh H. (2017). Cystine uptake through the cystine/glutamate antiporter xCT triggers glioblastoma cell death under glucose deprivation. J. Biol. Chem. 292 (48), 19721–19732. 10.1074/jbc.M117.814392 29038291 PMC5712613

[B51] González-Domínguez Á. Visiedo-García F. M. Domínguez-Riscart J. González-Domínguez R. Mateos R. M. Lechuga-Sancho A. M. (2020). Iron metabolism in obesity and metabolic syndrome. Int. J. Mol. Sci. 21 (15), 5529. 10.3390/ijms21155529 32752277 PMC7432525

[B52] Green D. R. Victor B. (2012). The pantheon of the fallen: why are there so many forms of cell death? Trends Cell Biol. 22 (11), 555–556. 10.1016/j.tcb.2012.08.008 22995729 PMC3568685

[B53] Guerrero-Hue M. García-Caballero C. Palomino-Antolín A. Rubio-Navarro A. Vázquez-Carballo C. Herencia C. (2019). Curcumin reduces renal damage associated with rhabdomyolysis by decreasing ferroptosis-mediated cell death. FASEB J. 33 (8), 8961–8975. 10.1096/fj.201900077R 31034781

[B54] Gui M. Farley H. Anujan P. Anderson J. R. Maxwell D. W. Whitchurch J. B. (2021). *De novo* identification of mammalian ciliary motility proteins using cryo-EM. Cell 184 (23), 5791–5806.e19. 10.1016/j.cell.2021.10.007 34715025 PMC8595878

[B55] Gutierrez-Mariscal F. M. de la Cruz-Ares S. Torres-Peña J. D. Alcalá-Diaz J. F. Yubero-Serrano E. M. López-Miranda J. (2021). Coenzyme Q(10) and cardiovascular diseases. Antioxidants (Basel, Switz.) 10 (6), 906. 10.3390/antiox10060906 34205085 PMC8229886

[B56] Hamza I. Prohaska J. Gitlin J. D. (2003). Essential role for Atox1 in the copper-mediated intracellular trafficking of the Menkes ATPase. Proc. Natl. Acad. Sci. U. S. A. 100 (3), 1215–1220. 10.1073/pnas.0336230100 12538877 PMC298753

[B57] Han S. Bao X. Zou Y. Wang L. Li Y. Yang L. (2023). d-lactate modulates M2 tumor-associated macrophages and remodels immunosuppressive tumor microenvironment for hepatocellular carcinoma. Sci. Adv. 9 (29), eadg2697. 10.1126/sciadv.adg2697 37467325 PMC10355835

[B58] Haq R. Shoag J. Andreu-Perez P. Yokoyama S. Edelman H. Rowe G. C. (2013). Oncogenic BRAF regulates oxidative metabolism *via* PGC1α and MITF. Cancer Cell 23 (3), 302–315. 10.1016/j.ccr.2013.02.003 23477830 PMC3635826

[B59] Hong X. Roh W. Sullivan R. J. Wong K. H. K. Wittner B. S. Guo H. (2021). The lipogenic regulator SREBP2 induces transferrin in circulating melanoma cells and suppresses ferroptosis. Cancer Discov. 11 (3), 678–695. 10.1158/2159-8290.Cd-19-1500 33203734 PMC7933049

[B60] Horng Y. C. Cobine P. A. Maxfield A. B. Carr H. S. Winge D. R. (2004). Specific copper transfer from the Cox17 metallochaperone to both Sco1 and Cox11 in the assembly of yeast cytochrome C oxidase. J. Biol. Chem. 279 (34), 35334–35340. 10.1074/jbc.M404747200 15199057

[B61] Hotchkiss R. S. Strasser A. McDunn J. E. Swanson P. E. (2009). Cell death. N. Engl. J. Med. 361 (16), 1570–1583. 10.1056/NEJMra0901217 19828534 PMC3760419

[B62] Hou G. Qian J. Wang Y. Xu W. Guo M. Li Z. (2023). Hydrazide/Metal/indocyanine green coordinated nanoplatform for potentiating reciprocal ferroptosis and immunity against melanoma. ACS Appl. Mater. Interfaces 15 (31), 37143–37156. 10.1021/acsami.3c05580 37498789

[B63] Hu B. Hounye A. H. Wang Z. Qi M. Zhang J. (2023). A novel Cuprotosis-related signature predicts the prognosis and selects personal treatments for melanoma based on bioinformatics analysis. Front. Oncol. 13, 1108128. 10.3389/fonc.2023.1108128 36824136 PMC9941880

[B64] Huang W. Yang F. Zhang Y. Fang Q. Lai Y. Lan Y. (2023). A newly established Cuproptosis-related gene signature for predicting prognosis and immune infiltration in uveal melanoma. Int. J. Mol. Sci. 24 (14), 11358. 10.3390/ijms241411358 37511120 PMC10379443

[B65] Huang Y. Chen C. Tan H. Dong S. Ren Y. Chao M. (2024). A stimulus-responsive ternary heterojunction boosting oxidative stress, cuproptosis for melanoma therapy. Small (Weinheim der Bergstrasse, Ger.) 20 (38), e2401147. 10.1002/smll.202401147 38770990

[B66] Huo S. Wang Q. Shi W. Peng L. Jiang Y. Zhu M. (2023). ATF3/SPI1/SLC31A1 signaling promotes cuproptosis induced by advanced glycosylation end products in diabetic myocardial injury. Int. J. Mol. Sci. 24 (2), 1667. 10.3390/ijms24021667 36675183 PMC9862315

[B67] Ibarra N. Pollitt A. Insall R. H. (2005). Regulation of actin assembly by SCAR/WAVE proteins. Biochem. Soc. Trans. 33 (Pt 6), 1243–1246. 10.1042/bst0331243 16246088

[B68] Jiang Z. Lim S. O. Yan M. Hsu J. L. Yao J. Wei Y. (2021). TYRO3 induces anti-PD-1/PD-L1 therapy resistance by limiting innate immunity and tumoral ferroptosis. J. Clin. Investigation 131 (8), e139434. 10.1172/jci139434 33855973 PMC8262501

[B69] Joly J. H. Delfarah A. Phung P. S. Parrish S. Graham N. A. (2020). A synthetic lethal drug combination mimics glucose deprivation-induced cancer cell death in the presence of glucose. J. Biol. Chem. 295 (5), 1350–1365. 10.1074/jbc.RA119.011471 31914417 PMC6996897

[B70] Kage F. Döring H. Mietkowska M. Schaks M. Grüner F. Stahnke S. (2022). Lamellipodia-like actin networks in cells lacking WAVE regulatory complex. J. Cell Sci. 135 (15), jcs260364. 10.1242/jcs.260364 35971979 PMC9511706

[B71] Kamphorst J. J. Chung M. K. Fan J. Rabinowitz J. D. (2014). Quantitative analysis of acetyl-CoA production in hypoxic cancer cells reveals substantial contribution from acetate. Cancer Metabolism 2, 23. 10.1186/2049-3002-2-23 25671109 PMC4322440

[B72] Kamphorst J. J. Nofal M. Commisso C. Hackett S. R. Lu W. Grabocka E. (2015). Human pancreatic cancer tumors are nutrient poor and tumor cells actively scavenge extracellular protein. Cancer Res. 75 (3), 544–553. 10.1158/0008-5472.Can-14-2211 25644265 PMC4316379

[B73] Kapralov A. A. Yang Q. Dar H. H. Tyurina Y. Y. Anthonymuthu T. S. Kim R. (2020). Redox lipid reprogramming commands susceptibility of macrophages and microglia to ferroptotic death. Nat. Chem. Biol. 16 (3), 278–290. 10.1038/s41589-019-0462-8 32080625 PMC7233108

[B74] Kapur P. Rakheja D. Roy L. C. Hoang M. P. (2005). Fatty acid synthase expression in cutaneous melanocytic neoplasms. Mod. Pathol. 18 (8), 1107–1112. 10.1038/modpathol.3800395 15920554

[B75] Khamari R. Trinh A. Gabert P. E. Corazao-Rozas P. Riveros-Cruz S. Balayssac S. (2018). Glucose metabolism and NRF2 coordinate the antioxidant response in melanoma resistant to MAPK inhibitors. Cell Death Dis. 9 (3), 325. 10.1038/s41419-018-0340-4 29487283 PMC5832419

[B76] Kim S. E. Zhang L. Ma K. Riegman M. Chen F. Ingold I. (2016). Ultrasmall nanoparticles induce ferroptosis in nutrient-deprived cancer cells and suppress tumour growth. Nat. Nanotechnol. 11 (11), 977–985. 10.1038/nnano.2016.164 27668796 PMC5108575

[B77] Koppula P. Zhang Y. Zhuang L. Gan B. (2018). Amino acid transporter SLC7A11/xCT at the crossroads of regulating redox homeostasis and nutrient dependency of cancer. Cancer Commun. Lond. Engl. 38 (1), 12. 10.1186/s40880-018-0288-x 29764521 PMC5993148

[B78] Koundouros N. Poulogiannis G. (2020). Reprogramming of fatty acid metabolism in cancer. Br. J. cancer 122 (1), 4–22. 10.1038/s41416-019-0650-z 31819192 PMC6964678

[B79] Kraft V. A. N. Bezjian C. T. Pfeiffer S. Ringelstetter L. Müller C. Zandkarimi F. (2020). GTP cyclohydrolase 1/Tetrahydrobiopterin counteract ferroptosis through lipid remodeling. ACS Central Sci. 6 (1), 41–53. 10.1021/acscentsci.9b01063 31989025 PMC6978838

[B80] Kubota C. S. Espenshade P. J. (2022). Targeting Stearoyl-CoA desaturase in solid tumors. Cancer Res. 82 (9), 1682–1688. 10.1158/0008-5472.Can-21-4044 35294526 PMC9064960

[B81] Kung Y. A. Chiang H. J. Li M. L. Gong Y. N. Chiu H. P. Hung C. T. (2022). Acyl-coenzyme A synthetase long-chain family member 4 is involved in viral replication organelle formation and facilitates virus replication *via* ferroptosis. mBio 13 (1), e0271721. 10.1128/mbio.02717-21 35038927 PMC8764547

[B82] La Fontaine S. Mercer J. F. (2007). Trafficking of the copper-ATPases, ATP7A and ATP7B: role in copper homeostasis. Archives Biochem. Biophys. 463 (2), 149–167. 10.1016/j.abb.2007.04.021 17531189

[B83] Lakhter A. J. Hamilton J. Konger R. L. Brustovetsky N. Broxmeyer H. E. Naidu S. R. (2016). Glucose-independent acetate metabolism promotes melanoma cell survival and tumor growth. J. Biol. Chem. 291 (42), 21869–21879. 10.1074/jbc.M115.712166 27539851 PMC5063972

[B84] Lee J. Roh J. L. (2023). Targeting iron-sulfur clusters in cancer: opportunities and challenges for ferroptosis-based therapy. Cancers 15 (10), 2694. 10.3390/cancers15102694 37345031 PMC10216707

[B85] Lei Y. Wang L. Liu P. Song Y. Gong Y. Jiang Y. (2024). Clarifying new molecular subtyping and precise treatment of melanoma based on disulfidptosis-related lncRNA signature. Eur. J. Med. Res. 29 (1), 468. 10.1186/s40001-024-02035-8 39342368 PMC11438283

[B86] Li W. Zhang C. Du H. Huang V. Sun B. Harris J. P. (2016). Withaferin A suppresses the up-regulation of acetyl-coA carboxylase 1 and skin tumor formation in a skin carcinogenesis mouse model. Mol. Carcinog. 55 (11), 1739–1746. 10.1002/mc.22423 26472150

[B87] Li Q. Peng F. Yan X. Chen Y. Zhou J. Wu S. (2023). Inhibition of SLC7A11-GPX4 signal pathway is involved in aconitine-induced ferroptosis *in vivo* and *in vitro* . J. Ethnopharmacol. 303, 116029. 10.1016/j.jep.2022.116029 36503029

[B88] Li H. Chen Z. Huang Y. Chen C. Cai L. (2024). Establishing a ten disulfidptosis-related gene signature for prognostic prediction in skin cutaneous melanoma. Comb Chem High Throughput Screen 28, 1952–1964. 10.2174/0113862073307469240528065718 38879775

[B89] Li Q. Cui Y. Xia Z. Gao W. Xiao J. Zhao Z. (2025). Biodegradable nano-immune agonist for enhanced immunotherapy of melanoma *via* the synergistic action of cuproptosis and cGAS-STING enhanced immune response. J. Colloid Interface Sci. 690, 137326. 10.1016/j.jcis.2025.137326 40120375

[B90] Liao P. Wang W. Wang W. Kryczek I. Li X. Bian Y. (2022). CD8^+^ T cells and fatty acids orchestrate tumor ferroptosis and immunity *via* ACSL4. Cancer Cell 40 (4), 365–378.e6. 10.1016/j.ccell.2022.02.003 35216678 PMC9007863

[B91] Liu X. Olszewski K. Zhang Y. Lim E. W. Shi J. Zhang X. (2020). Cystine transporter regulation of pentose phosphate pathway dependency and disulfide stress exposes a targetable metabolic vulnerability in cancer. Nat. Cell Biol. 22 (4), 476–486. 10.1038/s41556-020-0496-x 32231310 PMC7194135

[B92] Liu M. R. Zhu W. T. Pei D. S. (2021). System Xc(-): a key regulatory target of ferroptosis in cancer. Investig. New Drugs 39 (4), 1123–1131. 10.1007/s10637-021-01070-0 33506324

[B93] Liu J. Y. Liu L. P. Li Z. Luo Y. W. Liang F. (2022). The role of cuproptosis-related gene in the classification and prognosis of melanoma. Front. Immunol. 13, 986214. 10.3389/fimmu.2022.986214 36341437 PMC9632664

[B94] Liu X. Nie L. Zhang Y. Yan Y. Wang C. Colic M. (2023a). Actin cytoskeleton vulnerability to disulfide stress mediates disulfidptosis. Nat. Cell Biol. 25 (3), 404–414. 10.1038/s41556-023-01091-2 36747082 PMC10027392

[B95] Liu D. Yang F. Zhang T. Mao R. (2023b). Leveraging a cuproptosis-based signature to predict the prognosis and drug sensitivity of cutaneous melanoma. J. Transl. Med. 21 (1), 57. 10.1186/s12967-023-03891-4 36717900 PMC9885583

[B96] Liu Y. Li S. Wu Y. Zhang P. Yu Y. Chen X. (2025). Molecular signatures of disulfidptosis: interplay with programmed cell death pathways and therapeutic implications in oncology. Cell. Mol. Biol. Lett. 30 (1), 66. 10.1186/s11658-025-00743-5 40457177 PMC12128263

[B97] Long G. V. Swetter S. M. Menzies A. M. Gershenwald J. E. Scolyer R. A. (2023). Cutaneous melanoma. Lancet (London, Engl.) 402 (10400), 485–502. 10.1016/s0140-6736(23)00821-8 37499671

[B98] Lőrincz T. Jemnitz K. Kardon T. Mandl J. Szarka A. (2015). Ferroptosis is involved in acetaminophen induced cell death. Pathology Oncol. Res. 21 (4), 1115–1121. 10.1007/s12253-015-9946-3 25962350

[B99] Lu H. Liang J. He X. Ye H. Ruan C. Shao H. (2023). A novel oncogenic role of FDX1 in human melanoma related to PD-L1 immune checkpoint. Int. J. Mol. Sci. 24 (11), 9182. 10.3390/ijms24119182 37298135 PMC10253061

[B100] Lu X. Chen X. Lin C. Yi Y. Zhao S. Zhu B. (2024). Elesclomol loaded copper oxide nanoplatform triggers cuproptosis to enhance antitumor immunotherapy. Adv. Sci. (Weinheim, Baden-Wurttemberg, Ger.) 11 (18), e2309984. 10.1002/advs.202309984 38430531 PMC11095170

[B101] Lui J. W. Moore S. P. G. Huang L. Ogomori K. Li Y. Lang D. (2022). YAP facilitates melanoma migration through regulation of actin-related protein 2/3 complex subunit 5 (ARPC5). Pigment Cell Melanoma Res. 35 (1), 52–65. 10.1111/pcmr.13013 34468072 PMC8958630

[B102] Luo X. Gong H. B. Gao H. Y. Wu Y. P. Sun W. Y. Li Z. Q. (2021). Oxygenated phosphatidylethanolamine navigates phagocytosis of ferroptotic cells by interacting with TLR2. Cell Death Differ. 28 (6), 1971–1989. 10.1038/s41418-020-00719-2 33432112 PMC8185102

[B103] Lutsenko S. Barnes N. L. Bartee M. Y. Dmitriev O. Y. (2007). Function and regulation of human copper-transporting ATPases. Physiol. Rev. 87 (3), 1011–1046. 10.1152/physrev.00004.2006 17615395

[B104] Lv H. Liu X. Zeng X. Liu Y. Zhang C. Zhang Q. (2022). Comprehensive analysis of Cuproptosis-related genes in immune infiltration and prognosis in melanoma. Front. Pharmacol. 13, 930041. 10.3389/fphar.2022.930041 35837286 PMC9273972

[B105] Lv H. Liu L. He Y. Yang K. Fu Y. Bao Y. (2024). Role of hippo pathway and cuproptosis-related genes in immune infiltration and prognosis of skin cutaneous melanoma. Front. Pharmacol. 15, 1344755. 10.3389/fphar.2024.1344755 38515849 PMC10955143

[B106] Mao C. Liu X. Zhang Y. Lei G. Yan Y. Lee H. (2021). DHODH-mediated ferroptosis defence is a targetable vulnerability in cancer. Nature 593 (7860), 586–590. 10.1038/s41586-021-03539-7 33981038 PMC8895686

[B107] Mao C. Wang M. Zhuang L. Gan B. (2024). Metabolic cell death in cancer: ferroptosis, cuproptosis, disulfidptosis, and beyond. Protein Cell 15 (9), 642–660. 10.1093/procel/pwae003 38428031 PMC11365558

[B108] Marmonti E. Oliva-Ramirez J. Haymaker C. (2022). Dendritic cells: the long and evolving road towards successful targetability in cancer. Cells 11 (19), 3028. 10.3390/cells11193028 36230990 PMC9563837

[B109] Marzagalli M. Ebelt N. D. Manuel E. R. (2019). Unraveling the crosstalk between melanoma and immune cells in the tumor microenvironment. Seminars Cancer Biol. 59, 236–250. 10.1016/j.semcancer.2019.08.002 31404607

[B110] Mashimo T. Pichumani K. Vemireddy V. Hatanpaa K. J. Singh D. K. Sirasanagandla S. (2014). Acetate is a bioenergetic substrate for human glioblastoma and brain metastases. Cell 159 (7), 1603–1614. 10.1016/j.cell.2014.11.025 25525878 PMC4374602

[B111] Mason K. E. (1979). A conspectus of research on copper metabolism and requirements of man. J. Nutr. 109 (11), 1979–2066. 10.1093/jn/109.11.1979 387922

[B112] Mi T. Kong X. Chen M. Guo P. He D. (2024). Inducing disulfidptosis in tumors:potential pathways and significance. MedComm 5 (11), e791. 10.1002/mco2.791 39415848 PMC11480524

[B113] Min Y. Mao C. Q. Chen S. Ma G. Wang J. Liu Y. (2012). Combating the drug resistance of cisplatin using a platinum prodrug based delivery system. Angewandte Chemie Int. ed Engl. 51 (27), 6742–6747. 10.1002/anie.201201562 22639083

[B114] Mohapatra D. Senapati P. C. Senapati S. Pandey V. Dubey P. K. Singh S. (2024). Quality-by-design-based microemulsion of disulfiram for repurposing in melanoma and breast cancer therapy. Ther. Deliv. 15 (7), 521–544. 10.1080/20415990.2024.2363136 38949622 PMC11412148

[B115] More S. Bonnereau J. Wouters D. Spotbeen X. Karras P. Rizzollo F. (2024). Secreted Apoe rewires melanoma cell state vulnerability to ferroptosis. Sci. Adv. 10 (42), eadp6164. 10.1126/sciadv.adp6164 39413195 PMC11808924

[B116] Nagane M. Kanai E. Shibata Y. Shimizu T. Yoshioka C. Maruo T. (2018). Sulfasalazine, an inhibitor of the cystine-glutamate antiporter, reduces DNA damage repair and enhances radiosensitivity in murine B16F10 melanoma. PLoS One 13 (4), e0195151. 10.1371/journal.pone.0195151 29649284 PMC5896924

[B117] Nie D. Chen C. Li Y. Zeng C. (2022). Disulfiram, an aldehyde dehydrogenase inhibitor, works as a potent drug against sepsis and cancer *via* NETosis, pyroptosis, apoptosis, ferroptosis, and cuproptosis. Blood Sci. (Baltim. Md) 4 (3), 152–154. 10.1097/bs9.0000000000000117 36518588 PMC9742096

[B118] Oppenheimer L. Wellner V. P. Griffith O. W. Meister A. (1979). Glutathione synthetase. Purification from rat kidney and mapping of the substrate binding sites. J. Biol. Chem. 254 (12), 5184–5190. 10.1016/s0021-9258(18)50577-9 447639

[B119] Palmgren M. G. Nissen P. (2011). P-type ATPases. Annu. Rev. Biophys. 40, 243–266. 10.1146/annurev.biophys.093008.131331 21351879

[B120] Parascandolo A. Laukkanen M. O. (2019). Carcinogenesis and reactive oxygen species signaling: interaction of the NADPH oxidase NOX1-5 and superoxide dismutase 1-3 signal transduction pathways. Antioxidants Redox Signal. 30 (3), 443–486. 10.1089/ars.2017.7268 29478325 PMC6393772

[B121] Pereira D. J. Schoolwerth A. C. Pais V. M. (2015). Cystinuria: current concepts and future directions. Clin. Nephrol. 83 (3), 138–146. 10.5414/cn108514 25685869

[B122] Pich C. Meylan P. Mastelic-Gavillet B. Nguyen T. N. Loyon R. Trang B. K. (2018). Induction of paracrine signaling in metastatic melanoma cells by PPARγ agonist rosiglitazone activates stromal cells and enhances tumor growth. Cancer Res. 78 (22), 6447–6461. 10.1158/0008-5472.Can-18-0912 30185551

[B123] Poznanski S. M. Singh K. Ritchie T. M. Aguiar J. A. Fan I. Y. Portillo A. L. (2021). Metabolic flexibility determines human NK cell functional fate in the tumor microenvironment. Cell Metab. 33 (6), 1205–1220.e5. 10.1016/j.cmet.2021.03.023 33852875

[B124] Prohaska J. R. Geissler J. Brokate B. Broderius M. (2003). Copper, zinc-superoxide dismutase protein but not mRNA is lower in copper-deficient mice and mice lacking the copper chaperone for superoxide dismutase. Exp. Biol. Med. (Maywood, NJ) 228 (8), 959–966. 10.1177/153537020322800812 12968068

[B125] Prosser B. L. Ward C. W. Lederer W. J. (2011). X-ROS signaling: rapid mechano-chemo transduction in heart. Sci. (New York, NY) 333 (6048), 1440–1445. 10.1126/science.1202768 21903813

[B126] Qiao S. Cabello C. M. Lamore S. D. Lesson J. L. Wondrak G. T. (2012). D-Penicillamine targets metastatic melanoma cells with induction of the unfolded protein response (UPR) and Noxa (PMAIP1)-dependent mitochondrial apoptosis. Apoptosis Int. J. Program. Cell Death. 17 (10), 1079–1094. 10.1007/s10495-012-0746-x 22843330 PMC3779642

[B127] Robert C. Grob J. J. Stroyakovskiy D. Karaszewska B. Hauschild A. Levchenko E. (2019). Five-year outcomes with dabrafenib plus trametinib in metastatic melanoma. N. Engl. J. Med. 381 (7), 626–636. 10.1056/NEJMoa1904059 31166680

[B128] Rosner M. H. Bolton W. K. (2009). Ferumoxytol for the treatment of anemia in chronic kidney disease. Drugs today (Barcelona, Spain 1998) 45 (11), 779–786. 10.1358/dot.2009.45.11.1420459 20126670

[B129] Rowland E. A. Snowden C. K. Cristea I. M. (2018). Protein lipoylation: an evolutionarily conserved metabolic regulator of health and disease. Curr. Opin. Chem. Biol. 42, 76–85. 10.1016/j.cbpa.2017.11.003 29169048 PMC5965299

[B130] Rudolf E. Rudolf K. (2021). Acute increases in intracellular zinc lead to an increased lysosomal and mitochondrial autophagy and subsequent cell demise in malignant melanoma. Int. J. Mol. Sci. 22 (2), 667. 10.3390/ijms22020667 33440911 PMC7826594

[B131] Schmidt K. Ralle M. Schaffer T. Jayakanthan S. Bari B. Muchenditsi A. (2018). ATP7A and ATP7B copper transporters have distinct functions in the regulation of neuronal dopamine-β-hydroxylase. J. Biol. Chem. 293 (52), 20085–20098. 10.1074/jbc.RA118.004889 30341172 PMC6311498

[B132] Schöckel L. Glasauer A. Basit F. Bitschar K. Truong H. Erdmann G. (2015). Targeting mitochondrial complex I using BAY 87-2243 reduces melanoma tumor growth. Cancer Metabolism 3, 11. 10.1186/s40170-015-0138-0 26500770 PMC4615872

[B133] Simmen F. A. Alhallak I. Simmen R. C. M. (2020). Malic enzyme 1 (ME1) in the biology of cancer: it is not just intermediary metabolism. J. Mol. Endocrinol. 65 (4), R77–r90. 10.1530/jme-20-0176 33064660 PMC7577320

[B134] Singer K. Kastenberger M. Gottfried E. Hammerschmied C. G. Büttner M. Aigner M. (2011). Warburg phenotype in renal cell carcinoma: high expression of glucose-transporter 1 (GLUT-1) correlates with low CD8^+^ T-cell infiltration in the tumor. Int. J. cancer 128 (9), 2085–2095. 10.1002/ijc.25543 20607826

[B135] Soula M. Weber R. A. Zilka O. Alwaseem H. La K. Yen F. (2020). Metabolic determinants of cancer cell sensitivity to canonical ferroptosis inducers. Nat. Chem. Biol. 16 (12), 1351–1360. 10.1038/s41589-020-0613-y 32778843 PMC8299533

[B136] Stater E. P. Morcos G. Isaac E. Ogirala A. Hsu H. T. Longo V. A. (2023). Translatable drug-loaded iron oxide nanophore sensitizes Murine melanoma tumors to monoclonal antibody immunotherapy. ACS Nano 17 (7), 6178–6192. 10.1021/acsnano.2c05800 36971591 PMC10324163

[B137] Stockwell B. R. (2022). Ferroptosis turns 10: emerging mechanisms, physiological functions, and therapeutic applications. Cell. 185 (14), 2401–2421. 10.1016/j.cell.2022.06.003 35803244 PMC9273022

[B138] Sui X. Zhang R. Liu S. Duan T. Zhai L. Zhang M. (2018). RSL3 drives ferroptosis through GPX4 inactivation and ROS production in colorectal cancer. Front. Pharmacol. 9, 1371. 10.3389/fphar.2018.01371 30524291 PMC6262051

[B139] Sun X. Ou Z. Chen R. Niu X. Chen D. Kang R. (2016). Activation of the p62-Keap1-NRF2 pathway protects against ferroptosis in hepatocellular carcinoma cells. Hepatol. (Baltim. Md) 63 (1), 173–184. 10.1002/hep.28251 26403645 PMC4688087

[B140] Sun Y. Lei S. Luo X. Jiang C. Li Z. (2023). The value of cuproptosis-related differential genes in guiding prognosis and immune status in patients with skin cutaneous melanoma. Front. Pharmacol. 14, 1129544. 10.3389/fphar.2023.1129544 37138850 PMC10149708

[B141] Ta N. Jiang X. Zhang Y. Wang H. (2023). Ferroptosis as a promising therapeutic strategy for melanoma. Front. Pharmacol. 14, 1252567. 10.3389/fphar.2023.1252567 37795022 PMC10546212

[B142] Tang D. Kang R. Berghe T. V. Vandenabeele P. Kroemer G. (2019). The molecular machinery of regulated cell death. Cell Res. 29 (5), 347–364. 10.1038/s41422-019-0164-5 30948788 PMC6796845

[B143] Tang D. Chen X. Kang R. Kroemer G. (2021). Ferroptosis: molecular mechanisms and health implications. Cell Res. 31 (2), 107–125. 10.1038/s41422-020-00441-1 33268902 PMC8026611

[B144] Tao R. Li Y. Gong S. Zhang Q. Zhu Z. (2025). Unveiling intricating roles and mechanisms of ferroptosis in melanoma. Biochimica Biophys. Acta Rev. Cancer 1880 (1), 189234. 10.1016/j.bbcan.2024.189234 39644939

[B145] Teixido C. Castillo P. Martinez-Vila C. Arance A. Alos L. (2021). Molecular markers and targets in melanoma. Cells 10 (9), 2320. 10.3390/cells10092320 34571969 PMC8469294

[B146] Tian H. Deng H. Liu X. Liu C. Zhang C. Leong K. W. (2025). A novel FTO-targeting nanodrug induces disulfidptosis and ameliorates the suppressive tumor immune environment to treat uveal melanoma. Biomaterials 319, 123168. 10.1016/j.biomaterials.2025.123168 40015005

[B147] Tsoi J. Robert L. Paraiso K. Galvan C. Sheu K. M. Lay J. (2018). Multi-stage differentiation defines melanoma subtypes with differential vulnerability to drug-induced iron-dependent oxidative stress. Cancer Cell 33 (5), 890–904.e5. 10.1016/j.ccell.2018.03.017 29657129 PMC5953834

[B148] Tsvetkov P. Coy S. Petrova B. Dreishpoon M. Verma A. Abdusamad M. (2022). Copper induces cell death by targeting lipoylated TCA cycle proteins. Sci. (New York, NY) 375 (6586), 1254–1261. 10.1126/science.abf0529 35298263 PMC9273333

[B149] Tyrell R. Antia C. Stanley S. Deutsch G. B. (2017). Surgical resection of metastatic melanoma in the era of immunotherapy and targeted therapy. Melanoma Manag. 4 (1), 61–68. 10.2217/mmt-2016-0018 30190905 PMC6094597

[B150] Tyurina Y. Y. Kapralov A. A. Tyurin V. A. Shurin G. Amoscato A. A. Rajasundaram D. (2023). Redox phospholipidomics discovers pro-ferroptotic death signals in A375 melanoma cells *in vitro* and *in vivo* . Redox Biol. 61, 102650. 10.1016/j.redox.2023.102650 36870109 PMC9996109

[B151] Ubellacker J. M. Tasdogan A. Ramesh V. Shen B. Mitchell E. C. Martin-Sandoval M. S. (2020). Lymph protects metastasizing melanoma cells from ferroptosis. Nature 585 (7823), 113–118. 10.1038/s41586-020-2623-z 32814895 PMC7484468

[B152] Vergani E. Beretta G. L. Aloisi M. Costantino M. Corno C. Frigerio S. (2022). Targeting of the lipid metabolism impairs resistance to BRAF kinase inhibitor in melanoma. Front. Cell Dev. Biol. 10, 927118. 10.3389/fcell.2022.927118 35912092 PMC9326082

[B153] Vivas-García Y. Falletta P. Liebing J. Louphrasitthiphol P. Feng Y. Chauhan J. (2020). Lineage-restricted regulation of SCD and fatty acid saturation by MITF controls melanoma phenotypic plasticity. Mol. Cell 77 (1), 120–137.e9. 10.1016/j.molcel.2019.10.014 31733993 PMC7137507

[B154] Walshe J. M. (2007). Wilson’s disease. Lancet (London, Engl.) 369 (9565), 902. 10.1016/s0140-6736(07)60438-3 17368141

[B155] Wang W. Green M. Choi J. E. Gijón M. Kennedy P. D. Johnson J. K. (2019). CD8(+) T cells regulate tumour ferroptosis during cancer immunotherapy. Nature 569 (7755), 270–274. 10.1038/s41586-019-1170-y 31043744 PMC6533917

[B156] Wang D. Tian Z. Zhang P. Zhen L. Meng Q. Sun B. (2023a). The molecular mechanisms of cuproptosis and its relevance to cardiovascular disease. Biomed. Pharmacother. = Biomedecine Pharmacother. 163, 114830. 10.1016/j.biopha.2023.114830 37150036

[B157] Wang J. Li S. Guo Y. Zhao C. Chen Y. Ning W. (2023b). Cuproptosis-related gene SLC31A1 expression correlates with the prognosis and tumor immune microenvironment in glioma. Funct. Integr. Genomics 23 (3), 279. 10.1007/s10142-023-01210-0 37610668 PMC10447603

[B158] Wang J. Wang M. Wu S. Zhu Y. Fan K. Chen Y. (2024a). Tumor suppressor BAP1 suppresses disulfidptosis through the regulation of SLC7A11 and NADPH levels. Oncogenesis 13 (1), 31. 10.1038/s41389-024-00535-0 39266549 PMC11393423

[B159] Wang S. Guo Q. Xu R. Lin P. Deng G. Xia X. (2024b). Correction: combination of ferroptosis and pyroptosis dual induction by triptolide nano-MOFs for immunotherapy of melanoma. J. Nanobiotechnol. 22 (1), 415. 10.1186/s12951-024-02624-z 39010055 PMC11247738

[B160] Watson M. J. Vignali P. D. A. Mullett S. J. Overacre-Delgoffe A. E. Peralta R. M. Grebinoski S. (2021). Metabolic support of tumour-infiltrating regulatory T cells by lactic acid. Nature 591 (7851), 645–651. 10.1038/s41586-020-03045-2 33589820 PMC7990682

[B161] Wedan R. J. Longenecker J. Z. Nowinski S. M. (2024). Mitochondrial fatty acid synthesis is an emergent central regulator of mammalian oxidative metabolism. Cell Metab. 36 (1), 36–47. 10.1016/j.cmet.2023.11.017 38128528 PMC10843818

[B162] Wei X. Huang Q. Huang J. Yu L. Chen J. (2023). Erastin induces ferroptosis in cervical cancer cells *via* Nrf2/HO-1 signaling pathway. Int. J. Immunopathol. Pharmacol. 37, 3946320231219348. 10.1177/03946320231219348 38031977 PMC10687934

[B163] Wu J. H. Cheng T. C. Zhu B. Gao H. Y. Zheng L. Chen W. X. (2023). Identification of cuproptosis-related gene SLC31A1 and upstream LncRNA-miRNA regulatory axis in breast cancer. Sci. Rep. 13 (1), 18390. 10.1038/s41598-023-45761-5 37884650 PMC10603161

[B164] Xu H. Ye D. Ren M. Zhang H. Bi F. (2021). Ferroptosis in the tumor microenvironment: perspectives for immunotherapy. Trends Mol. Med. 27 (9), 856–867. 10.1016/j.molmed.2021.06.014 34312075

[B165] Xu S. Y. Yin S. S. Wang L. Zhong H. Wang H. Yu H. Y. (2025). Insights into emerging mechanisms of ferroptosis: new regulators for cancer therapeutics. Cell Biol. Toxicol. 41 (1), 63. 10.1007/s10565-025-10010-0 40131564 PMC11937073

[B166] Yan B. Ai Y. Sun Q. Ma Y. Cao Y. Wang J. (2021). Membrane damage during ferroptosis is caused by oxidation of phospholipids catalyzed by the oxidoreductases POR and CYB5R1. Mol. Cell 81 (2), 355–369.e10. 10.1016/j.molcel.2020.11.024 33321093

[B167] Yang Y. Luo M. Zhang K. Zhang J. Gao T. Connell D. O. (2020). Nedd4 ubiquitylates VDAC2/3 to suppress erastin-induced ferroptosis in melanoma. Nat. Commun. 11 (1), 433. 10.1038/s41467-020-14324-x 31974380 PMC6978386

[B168] Yang X. Wang X. Sun X. Xiao M. Fan L. Su Y. (2022). Construction of five cuproptosis-related lncRNA signature for predicting prognosis and immune activity in skin cutaneous melanoma. Front. Genet. 13, 972899. 10.3389/fgene.2022.972899 36160015 PMC9490379

[B169] Yang Y. Li Q. Chen J. Guo Y. Cai Y. Zhao W. (2024). A cuproptosis-related prognostic signature for guiding clinical diagnosis and treatment in uveal melanoma patients. Heliyon 10 (16), e36324. 10.1016/j.heliyon.2024.e36324 39247274 PMC11378888

[B170] Yao F. Cui X. Zhang Y. Bei Z. Wang H. Zhao D. (2021). Iron regulatory protein 1 promotes ferroptosis by sustaining cellular iron homeostasis in melanoma. Oncol. Lett. 22 (3), 657. 10.3892/ol.2021.12918 34386079 PMC8299017

[B171] Yao H. Liu P. Yao L. Li X. (2024). Establishment of disulfidptosis-related LncRNA signature as biomarkers in colon adenocarcinoma. Cancer Cell Int. 24 (1), 183. 10.1186/s12935-024-03374-6 38802854 PMC11131243

[B172] Yu Y. Ren Y. Wang C. Li Z. Niu F. Li Z. (2022). Arginase 2 negatively regulates sorafenib-induced cell death by mediating ferroptosis in melanoma. Acta Biochim. Biophys. Sin. 54 (11), 1658–1670. 10.3724/abbs.2022166 36604146 PMC9828469

[B173] Zang X. He X. Y. Xiao C. M. Lin Q. Wang M. Y. Liu C. Y. (2024). Circular RNA-encoded oncogenic PIAS1 variant blocks immunogenic ferroptosis by modulating the balance between SUMOylation and phosphorylation of STAT1. Mol. Cancer 23 (1), 207. 10.1186/s12943-024-02124-6 39334380 PMC11438063

[B174] Zarrinpar A. (2022). A high-protein diet prevents weight regain. Nat. Metab. 4 (12), 1616–1617. 10.1038/s42255-022-00699-2 36456725

[B175] Zhang D. Li J. Wang F. Hu J. Wang S. Sun Y. (2014). 2-Deoxy-D-glucose targeting of glucose metabolism in cancer cells as a potential therapy. Cancer Lett. 355 (2), 176–183. 10.1016/j.canlet.2014.09.003 25218591

[B176] Zhang L. Li X. M. Shi X. H. Ye K. Fu X. L. Wang X. (2023a). Sorafenib triggers ferroptosis *via* inhibition of HBXIP/SCD axis in hepatocellular carcinoma. Acta Pharmacol. Sin. 44 (3), 622–634. 10.1038/s41401-022-00981-9 36109580 PMC9958095

[B177] Zhang R. Kang R. Tang D. (2023b). Reductive cell death: the other side of the coin. Cancer Gene Ther. 30 (7), 929–931. 10.1038/s41417-023-00612-3 37016143

[B178] Zhang X. Tang B. Luo J. Yang Y. Weng Q. Fang S. (2024). Cuproptosis, ferroptosis and PANoptosis in tumor immune microenvironment remodeling and immunotherapy: culprits or new hope. Mol. Cancer 23 (1), 255. 10.1186/s12943-024-02130-8 39543600 PMC11566504

[B179] Zhang Y. Xie W. Li J. Liang Z. Zhou X. Tan Z. (2025). Precision targeted melanoma therapy *via* cuproptosis/chemodynamic and chemotherapy: an engineering MCHS-CuMOF nanodelivery system. Biomater. Adv. 171, 214228. 10.1016/j.bioadv.2025.214228 39983499

[B188] Zhang Y. Huang J. Huang Y. Zhang S. Wu W. Long H. (2021). Tanshinone’ I and simvastatin inhibit melanoma tumour cell growth by regulating poly (ADP ribose) polymerase’ 1 expression. Mol. Med. Rep. 23 (1). 10.3892/mmr.2020.11678 33179075 PMC7684874

[B180] Zhao B. Li X. Wang Y. Shang P. (2018). Iron-dependent cell death as executioner of cancer stem cells. J. Exp. Clin. Cancer Res. 37 (1), 79. 10.1186/s13046-018-0733-3 29636068 PMC5894200

[B181] Zhao Y. Wei Y. Fan L. Nie Y. Li J. Zeng R. (2023). Leveraging a disulfidptosis-related signature to predict the prognosis and immunotherapy effectiveness of cutaneous melanoma based on machine learning. Mol. Med. (Camb. Mass) 29 (1), 145. 10.1186/s10020-023-00739-x 37884883 PMC10601311

[B182] Zheng P. Zhou C. Lu L. Liu B. Ding Y. (2022). Elesclomol: a copper ionophore targeting mitochondrial metabolism for cancer therapy. J. Exp. Clin. Cancer Res. 41 (1), 271. 10.1186/s13046-022-02485-0 36089608 PMC9465867

[B183] Zhou J. Liu S. Wang Y. Dai W. Zou H. Wang S. (2019). Salinomycin effectively eliminates cancer stem-like cells and obviates hepatic metastasis in uveal melanoma. Mol. Cancer 18 (1), 159. 10.1186/s12943-019-1068-1 31718679 PMC6852970

[B184] Zhou Y. Shu Q. Fu Z. Wang C. Gu J. Li J. (2022). A novel risk model based on cuproptosis-related lncRNAs predicted prognosis and indicated immune microenvironment landscape of patients with cutaneous melanoma. Front. Genet. 13, 959456. 10.3389/fgene.2022.959456 35938036 PMC9354044

[B185] Zhou B. Sha S. Wang Q. Sun S. Tao J. Zhu J. (2024). The prognostic implications of cuproptosis-related gene signature and the potential of PPIC as a promising biomarker in cutaneous melanoma. Pigment Cell Melanoma Res. 37 (6), 864–880. 10.1111/pcmr.13185 39115044

[B186] Zhu L. Kang X. Zhu S. Wang Y. Guo W. Zhu R. (2024). Cuproptosis-related DNA methylation signature predict prognosis and immune microenvironment in cutaneous melanoma. Discov. Oncol. 15 (1), 228. 10.1007/s12672-024-01089-8 38874871 PMC11178724

[B187] Zou Y. Palte M. J. Deik A. A. Li H. Eaton J. K. Wang W. (2019). A GPX4-dependent cancer cell state underlies the clear-cell morphology and confers sensitivity to ferroptosis. Nat. Commun. 10 (1), 1617. 10.1038/s41467-019-09277-9 30962421 PMC6453886

